# Endothelial PDGF Signaling Dysregulation Impairs Testicular Interstitial Homeostasis in Diabetes

**DOI:** 10.1002/advs.202520114

**Published:** 2026-02-05

**Authors:** Wenxiu Zhang, Kai Hong, Yanling Tang, Lina Cui, Xiaojian Lu, Jianxing Cheng, Yangyi Fang, Qiaoling Jiang, Ziyan Zhuang, Songzhan Gao, Hui Jiang, Qiang Liu, Jingtao Guo, Zhe Zhang, Xiaoyan Wang

**Affiliations:** ^1^ State Key Laboratory of Organ Regeneration and Reconstruction Institute of Zoology Chinese Academy of Sciences Beijing China; ^2^ Beijing Institute for Stem Cell and Regenerative Medicine Beijing China; ^3^ University of Chinese Academy of Sciences Beijing China; ^4^ Department of Urology Center for Reproductive Medicine State Key Laboratory of Female Fertility Promotion Peking University Third Hospital Beijing China; ^5^ Department of Andrology Third Affiliated Hospital of Zhengzhou University Zhengzhou Henan China; ^6^ Department of Urology Peking University First Hospital Beijing China; ^7^ The Institution of Urology Peking University Beijing China; ^8^ Beijing Key Laboratory of Urogenital Diseases (Male) Molecular Diagnosis and Treatment Center Beijing China; ^9^ State Key Laboratory of Female Fertility Promotion Center for Reproductive Medicine Department of Obstetrics and Gynecology Peking University Third Hospital Beijing China

**Keywords:** JUND, leydig cells, PDGF signaling, single‐cell RNA‐seq, testicular endothelial cells

## Abstract

The testicular interstitium relies on coordinated signaling among vascular, steroidogenic, and structural cells, yet the regulatory role of testicular endothelial cells (TECs) in maintaining this homeostasis remains unclear. Here, we identify TECs as a central signaling hub that orchestrates intercellular communication within the human testis. Integrative single‐cell transcriptomic analysis of healthy and diabetic testes reveals that diabetes disrupts platelet‐derived growth factor (PDGF) signaling. TECs in diabetes undergo endothelial‐to‐mesenchymal transition and exhibit reduced *PDGFB* expression, while Leydig and testicular peritubular cells downregulate *PDGFRB*, collectively weakening intercellular connectivity. This disruption silences the JUND‐MCL1 survival program in Leydig cells, leading to apoptosis, extracellular matrix accumulation, and testosterone insufficiency, while impairing the contractility of testicular peritubular cells. Importantly, exogenous PDGF‐BB supplementation reactivates the JUND‐MCL1 axis, protects Leydig cells, alleviates fibrosis, and partially restores testosterone production and peritubular function. Together, these findings establish endothelial PDGF dysregulation as a key driver of diabetic testicular pathology and highlight PDGF‐BB supplementation as a mechanistically grounded therapeutic strategy to restore interstitial and endocrine function in the context of diabetes.

## Graphical abstract

Testicular endothelial cells (TECs) function as central signaling hubs that coordinate interstitial homeostasis. Diabetes disrupts TEC‐derived PDGF signaling, silences the JUND‐MCL1 survival program in Leydig cells, and impairs peritubular function, leading to fibrosis and testosterone insufficiency. Exogenous PDGF‐BB supplementation restores intercellular communication and endocrine activity, revealing a mechanistically grounded therapeutic strategy to mitigate diabetic testicular dysfunction.

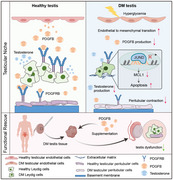

## 1 Introduction

Testicular function relies on a highly specialized interstitial microenvironment in which germ and somatic cells coordinate to sustain both spermatogenesis and endocrine activity [1]. Within this niche, testicular endothelial cells (TECs) have long been regarded as passive vascular scaffolds, and their potential regulatory roles have therefore remained underexplored. However, endothelial cells are increasingly recognized as heterogeneous and organ‐specific regulators of tissue homeostasis [2, 3, 4]. By extension, TECs may also exert unique paracrine functions within the testis. Indeed, recent work revealed that TECs secrete glial cell line‐derived neurotrophic factor to support spermatogonial stem cell maintenance, underscoring their active contribution to testicular homeostasis [5]. Still, the broader regulatory functions of TECs in the adult human testis remain poorly understood.

Diabetes mellitus (DM) is among the most prevalent metabolic disorders and is defined by systemic vascular injury and endothelial dysfunction [6]. The testis is no exception. Clinical and experimental studies consistently report microvascular disruption, including reduced vascular blood flow, vascular rarefaction, and endothelial abnormalities [7, 8]. These observations highlight the increased vulnerability of the testicular vasculature to metabolic stress, positioning diabetes as an ideal model for investigating how TEC dysfunction affects male reproductive function. Notably, unlike many forms of severe spermatogenic failure that are accompanied by extensive disruption of seminiferous architecture, diabetes impairs sperm morphology while largely preserving the organizational framework of spermatogenesis. Crucially, evidence from human tissue implicates dysregulated Apelin/APJ signaling in Sertoli cells as a key disruptor of the blood‐testis barrier, a mechanism that underpins the resultant decline in sperm quality [9]. However, due to the selective design of the Smart‐seq2 dataset, available findings are mainly limited to Sertoli cells, leaving other somatic populations insufficiently characterized.

Clinically, the most consistent reproductive consequence of diabetes is hypogonadism. Beyond reduced fertility, testosterone deficiency affects metabolism, bone health, cognition, and cardiovascular function [10, 11, 12, 13, 14]. In animal models, diabetes suppresses steroidogenic gene expression and induces stress responses in Leydig cells (LCs), leading to cell death and loss of steroidogenic capacity [15, 16]. However, these findings largely capture downstream events, and their direct relevance to humans is uncertain due to species differences. Current treatment strategies remain restricted to testosterone replacement therapy, which offers temporary relief but fails to address underlying mechanisms. Diabetes also compromises testicular peritubular cells (TPCs), reducing their contractile and supportive capacity [17]. These findings suggest that diabetes‐associated testicular dysfunction reflects a broader imbalance in the interstitial microenvironment, rather than being confined to a single cell type. However, it remains unclear whether the dysfunction of LCs and TPCs is independent or driven by a common upstream regulator.

Given their vascular position and signaling potential, TECs represent compelling candidates for mediating the effects of systemic metabolic stress on the testicular interstitium. Here, we profiled TECs from healthy and diabetic human testes using integrative single‐cell transcriptomics. We found that diabetes disrupts testicular interstitial homeostasis by impairing TEC‐derived platelet‐derived growth factor (PDGF) signaling, characterized by reduced *PDGFB* expression in TECs and downregulation of *PDGFRB* in LCs and TPCs. This disruption silences the JUND‐MCL1 survival program, driving LC apoptosis, extracellular matrix accumulation, and testosterone insufficiency. Importantly, *ex vivo* PDGF‐BB supplementation reactivated this pathway, restored the function of LCs, and alleviated interstitial fibrosis. Collectively, these findings identify TEC dysfunction as a central driver of diabetic testicular pathology and propose PDGF‐BB restoration as a mechanistically grounded therapeutic strategy to preserve interstitial and endocrine function.

## 2 Results

### 2.1 TECs Undergo Signaling Suppression and Transcriptional Alterations in Diabetes

**FIGURE 1:**
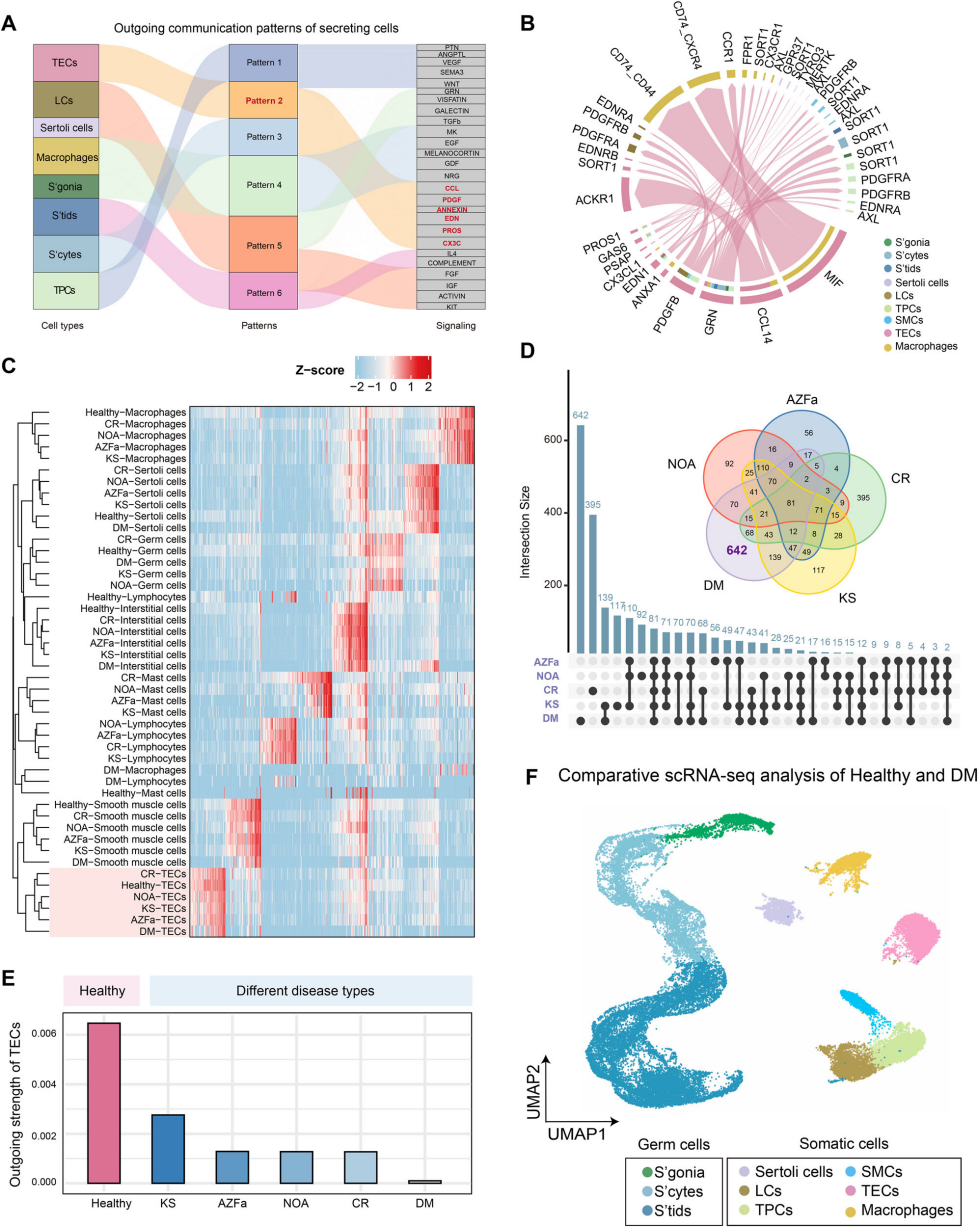
TECs exhibit signaling suppression and transcriptomic alterations in diabetes. (A) Sankey diagram providing an overview of the outgoing communication patterns (middle) of each type of secreting testicular cells (left) with the corresponding signaling pathways (right). (B) Circle plots displaying the signaling pathways derived from TECs to other testicular cells. The width of each edge corresponds to the number of unique ligand‐receptor pairs inferred between the two cell types. (C) Heatmap presenting the expression patterns of testicular cells in healthy and different disease conditions. Expression values are scaled to Z scores, with colors indicating relative expression levels. (D) UpSet plot visualizing intersections of differentially expressed genes (DEGs) in TECs from various disease states compared to healthy controls. The vertical bar chart (top) indicates the size of the gene set intersection for the specific combination of phenotypes connected below. The Venn plot (upper right) represents similar information. (E) Bar plot showing the outgoing signaling strength of TECs in healthy testes and in different disease types. Bar height denotes the summed outgoing signaling strength of TECs in each condition. (F) UMAP plot showing the major cell types in testicular samples. UMAP visualization of 38,758 cells derived from testicular samples of diabetes mellitus (DM, n = 5) patients and healthy adult males (healthy, n = 5). Each dot represents a single cell, and colors indicate distinct cell populations.

A previous study has suggested that testicular endothelial cells (TECs) play critical functions in sustaining testicular homeostasis in mice [5]. However, their regulatory functions in the human testis remain poorly defined. To address this gap, we integrated and reanalyzed publicly available single‐cell transcriptomic datasets of healthy human testes and mapped the outgoing signaling networks across cell types. Cell‐cell communication analysis revealed that healthy TECs exhibit multi‐pattern ligand‐receptor signaling and engage in extensive interactions with other cell populations (Figure 1A,B; Figure S1A). Quantitative assessment further showed that TECs ranked second in total outgoing signaling strength among all testicular cell types, second only to TPCs (Figure S1B), underscoring their pivotal regulatory role in testicular homeostasis.

To determine how TECs are altered under disease conditions, we analyzed published single‐cell transcriptomic datasets from male reproductive disorders, including Klinefelter syndrome (KS), azoospermia factor a deletion (hereafter referred to as AZFa), non‐obstructive azoospermia (NOA), congenital cryptorchidism (CR), and diabetes mellitus (DM). Although the DM dataset was highly informative, TECs were not captured due to the inherent bias of the Smart‐seq 2 platform [9]. To overcome this limitation, we generated unbiased droplet‐based 10x Genomics single‐cell transcriptomes from testicular tissues of three DM patients. After quality control, a total of 31,866 cells were retained for downstream analysis (Figure S1C). TECs were successfully recovered across healthy and all disease types, thereby enabling focused analyses of TECs (Figure 1C). Differential expression analysis revealed that TECs in each disease exhibited molecular alterations, with DM showing the greatest number of differentially expressed genes (DEGs) and the most pronounced global transcriptional changes compared with healthy controls (Figure 1D,E). These results suggested that diabetes represents the condition with the most profound TEC transcriptional alterations, establishing DM as an ideal pathological model to interrogate TEC dysfunction and its impact on the testicular microenvironment. Building on this insight, we next integrated the healthy control and DM datasets, removed batch effects, and constructed a unified single‐cell atlas (Figure S1D). Based on canonical marker expression, we identified nine major testicular cell types, and cell type annotations were further supported by Gene Ontology (GO) enrichment analysis of DEGs (Figure S1E,F; Figure 1F). Next, we compared transcriptional alterations across testicular cell types and observed that somatic cells, especially TECs, showed pronounced transcriptional changes and variability in DM, highlighting their vulnerability to diabetic stress (Figure S1G–I).

Collectively, we provide a comprehensive map of TEC signaling networks in the healthy human testis and uncover diabetes as the context in which TECs display the most extensive molecular alterations. These findings indicate that TECs may serve as an entry point for investigating the impact of metabolic stress on male reproduction.

### 2.2 Diabetes‐Induced Mesenchymal Transition and Angiogenic Impairment in TECs

**FIGURE 2:**
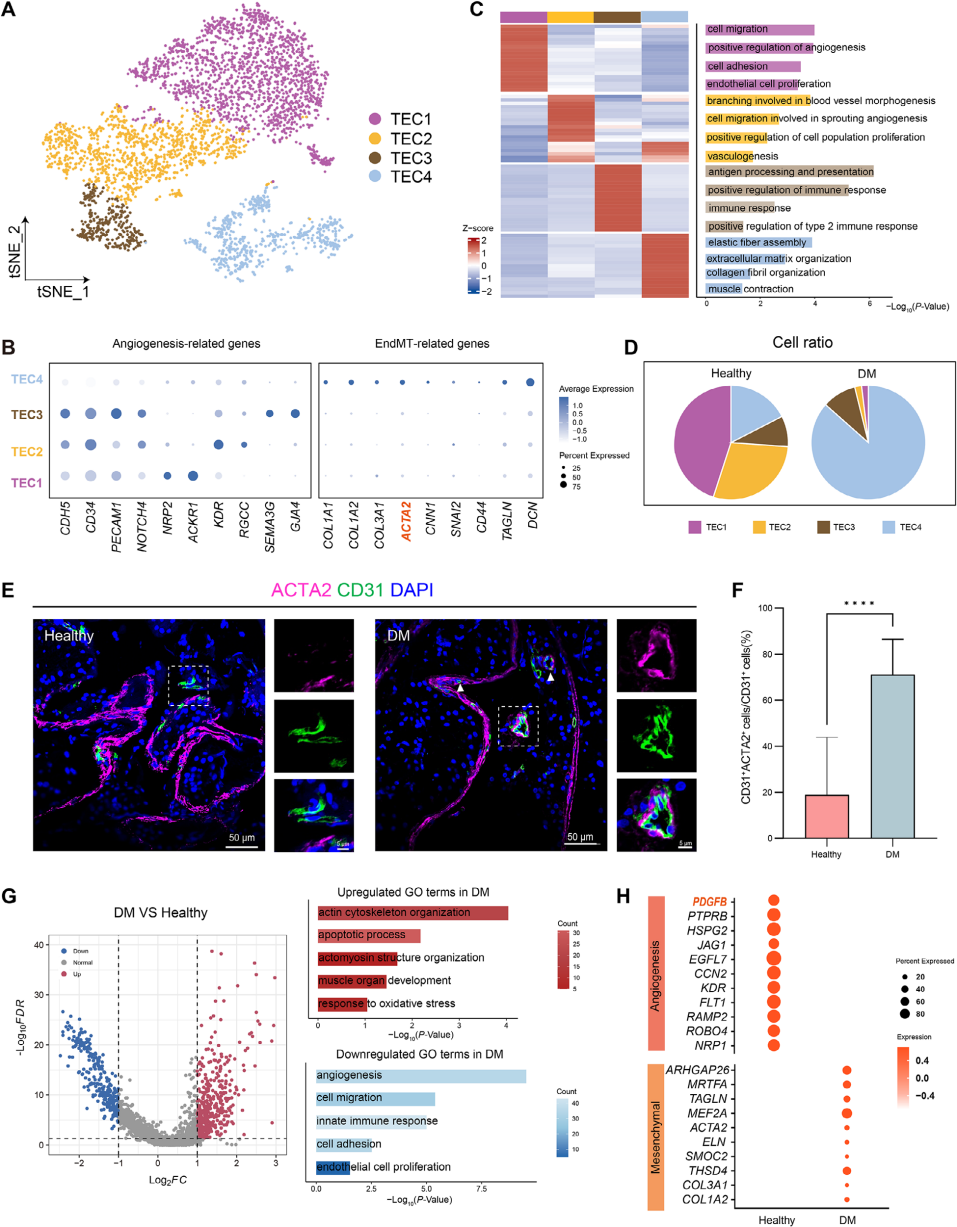
Deteriorated functions of angiogenesis and mesenchymal transition in diabetic TECs. (A) A t‐SNE plot showing the annotated TECs derived from the dataset in Figure 1F. Each dot represents a single cell, and colors denote distinct TEC subclusters. (B) Dot plot showing expression of angiogenesis‐related and EndMT‐related genes across TEC subclusters. The x‐axis lists genes and the y‐axis indicates TEC subclusters. Dot size indicates the proportion of cells expressing each gene, and color intensity represents the average expression level. (C) Left: Heatmap showing DEGs for each TECs subcluster. Expression values are scaled to Z scores, with colors indicating relative expression levels. Right: Bar plot of Gene Ontology (GO) terms significantly enriched in the marker genes of each cluster. Bar length represents –Log_10_(*p*‐value), reflecting the significance of enrichment. (D) Pie chart illustrating the relative proportions of TEC subclusters in healthy and DM testes. Each segment represents one TECs subcluster. (E) Representative immunofluorescence staining of ACTA2 (magenta) and CD31 (green) in testicular sections from healthy controls and diabetic patients. Nuclei are counterstained with DAPI (blue). Insets highlight higher magnification views of the boxed regions. Scale bars, 50 µm; 5 µm (insets). (F) Quantification of ACTA2^+^ CD31^+^ TECs among total CD31^+^ cells in healthy and DM testes. The x‐axis shows sample groups, and the y‐axis indicates the percentage of ACTA2^+^ CD31^+^ cells. Bars represent the mean ± standard deviation (SD). n = 3 independent biological replicates per group. Statistical significance was determined using the two‐tailed Student's t‐test. ****p < 0.0001. (G) Left: Volcano plot of differentially expressed genes (DEGs) in TECs between DM and healthy testes. Right: Bar plot of GO enrichment analysis of DEGs in DM TECs. Bar length represents the number of genes in each term, and color intensity indicates statistical significance –Log_10_(*p*‐value). (H) Dot plot showing genes associated with angiogenesis and mesenchymal transition in TECs from healthy and DM testes. Dot size indicates the proportion of cells expressing each gene, and color intensity represents the average expression level.

To further dissect the molecular alterations of TECs under diabetic conditions, we performed subcluster analysis of TECs and identified four transcriptionally distinct TEC subpopulations (TEC1‐TEC4) (Figure 2A). TEC1 and TEC2 were marked by high expression of *ACKR1* [18, 19, 20] and *RGCC* [2, 21] (Figure 2B; Figure S2A), and functional enrichment analysis revealed significant enrichment of vascular homeostasis and angiogenic pathways, including cell migration, positive regulation of angiogenesis, and vasculogenesis, consistent with venous and capillary endothelial identities [18, 22] (Figure 2C). TEC3 represents an arteriovenous transitional endothelial subpopulation, characterized by high expression of *SEMA3G* and *GJA4* [23]. This cluster was also enriched for immune regulatory processes, such as antigen processing and presentation and immune response, suggesting a potential role at the vascular‐immune interface of the testis (Figure 2C). TEC4 transcriptionally diverged from the other clusters and showed upregulation of extracellular matrix and mesenchymal‐associated genes (e.g., *COL1A1*, *COL3A1*, *ACTA2*, *CNN1*, *SNAI2*, *TAGLN*) (Figure 2B; Figure S2B), with enrichment of extracellular matrix organization, collagen fibril organization, and muscle contraction (Figure 2C), indicating a canonical endothelial‐to‐mesenchymal transition (EndMT) phenotype [24]. Despite these changes, TEC4 expressed canonical endothelial markers *VWF* and *CD34* (Figure S2C). TEC4 exhibited comparable transcript counts and gene detection to other subpopulations (Figure S2C), ruling out sequencing bias and confirming its identity as a bona fide endothelial lineage undergoing pathological mesenchymal transition. Notably, TEC4 displayed a transcriptional pattern resembling that previously reported in adipose tissue endothelial cells [25].

Compared with healthy controls, the proportion of TEC4 was increased in DM (Figure 2D). Immunofluorescence further confirmed an increased abundance of CD31^+^ ACTA2^+^ double‐positive cells in diabetic testes (Figure 2E,F; Figure S2D), suggesting that EndMT‐type TECs are expanded under diabetic conditions. Transcriptomic profiling further revealed upregulation of mesenchymal‐associated genes (e.g., *COL1A2*, *TAGLN*, *ACTA2*) and concomitant downregulation of angiogenic factors (*PDGFB*, *KDR*, *FLT1*, *NRP1*) in DM TECs (Figure 2H). These alterations were accompanied by suppression of pathways related to vascular homeostasis, migration, and proliferation, while stress responses, fibrotic remodeling, and cytoskeletal reorganization programs were activated (Figure 2G). Notably, DM TECs exhibited the lowest angiogenic potential among the pathological conditions analyzed (Figure S2E), underscoring the severe vascular dysfunction associated with diabetes.

Finally, it should be noted that TECs represent the principal source of *PDGFB* expression in the healthy testis (Figure S2F), thereby establishing the PDGF‐PDGFR signaling axis for the regulation of testicular homeostasis (Figure 1A,B; Figure S1A). In contrast, in diabetic TECs, *PDGFB* expression was reduced (Figure 2H; Figure S2G), suggesting attenuation of TEC‐derived PDGF signaling and offering an important clue for exploring diabetes‐associated testicular dysfunction.

### 2.3 Dual‐Level Dysfunction of TEC‐Derived PDGF Signaling in Diabetes

**FIGURE 3:**
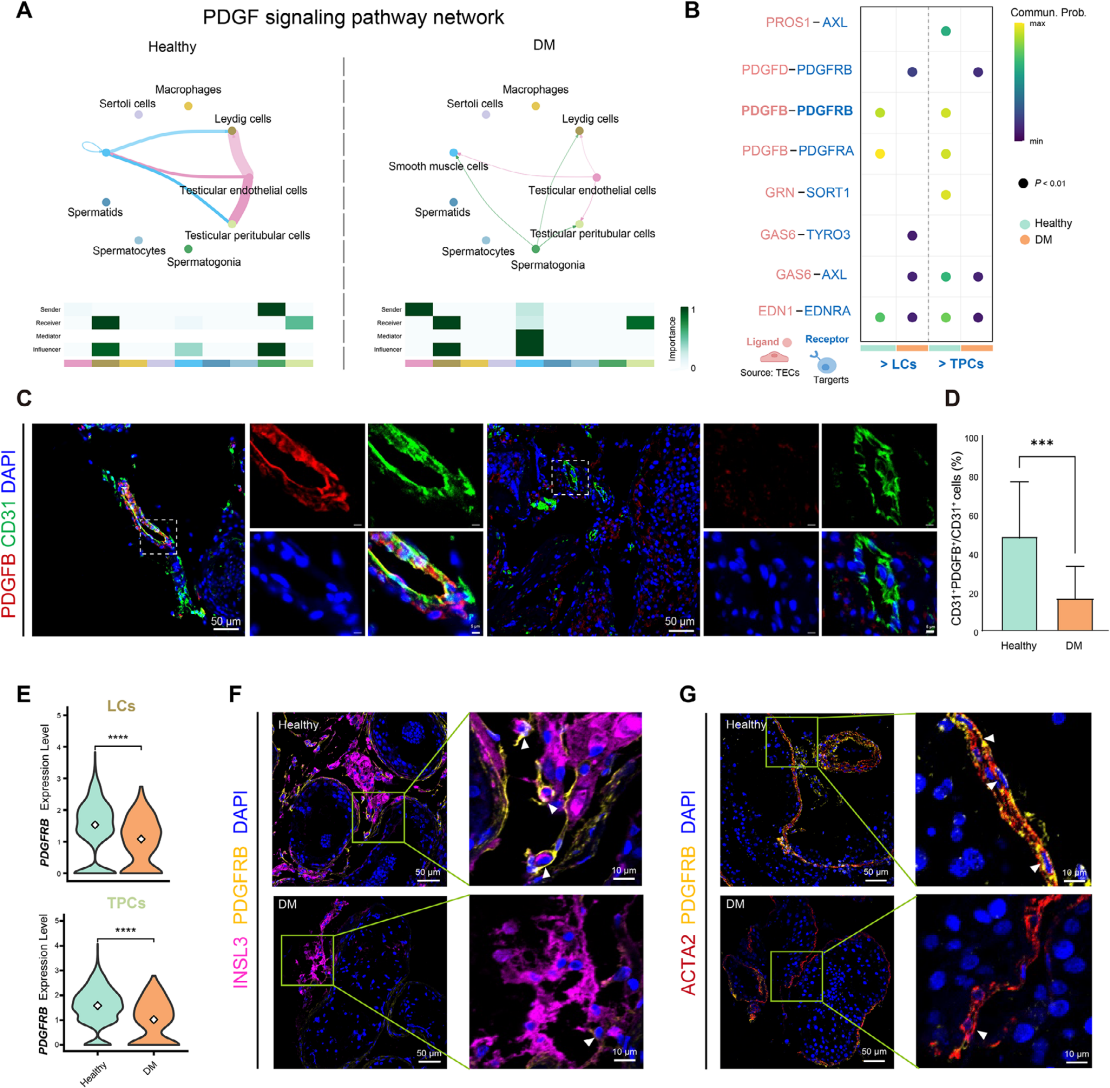
Impaired PDGFB‐PDGFRB signaling pathway from TECs to LCs and TPCs. (A) Top: Circle plots showing PDGF signaling network in healthy and DM testes. Each node represents a testicular cell type, and edges denote signaling interactions. Edge width is proportional to communication probability. Down: Heatmap showing the relative contributions of testicular cell types to PDGF signaling in healthy and DM testes. The x‐axis indicates cell types, and the y‐axis represents signaling roles (sender, receiver, mediator, and influencer). Color intensity reflects relative contribution scores. (B) Dot plot showing ligand and receptor interactions from TECs to LCs and TPCs. Dot color indicates communication probability, dot size reflects statistical significance (*p* < 0.01), and colors distinguish healthy and DM conditions. (C) Representative immunofluorescence staining of PDGFB (red) and CD31 (green) in testicular sections from healthy controls and diabetic patients. Nuclei are counterstained with DAPI (blue). Insets highlight higher magnification views of the boxed regions. Scale bars, 50 µm; 5 µm (insets). (D) Quantification of CD31^+^ PDGFB^+^ TECs expressed as a percentage of total CD31^+^ TECs. Data are presented as mean ± SD. n = 3 independent biological replicates per group. Statistical significance was determined using the two‐tailed Student's t‐test. ***p < 0.001. (E) Violin plots illustrating *PDGFRB* expression in LCs (top) and TPCs (bottom) from healthy and DM testes. ****p < 0.0001. (F) Representative immunofluorescence staining of PDGFRB (yellow), INSL3 (magenta), and nuclei (DAPI, blue) in testicular sections from healthy controls and DM patients. Insets highlight higher magnification views of the boxed regions. Scale bars, 50 µm; 10 µm (insets). (G) Representative immunofluorescence staining of PDGFRB (yellow), ACTA2 (red), and DAPI (blue) in testicular sections from healthy and DM patients. Insets show higher magnification of boxed regions. Scale bars, 50 µm; 10 µm (insets).

We next systematically profiled ligand‐receptor interactions between TECs and other testicular cell populations under healthy and diabetic conditions. Global interaction mapping revealed a marked reduction in TEC‐mediated interactions in diabetes (Figure S3A), indicating a broad attenuation of signaling input from TECs. Building on the observed transcriptional downregulation of *PDGFB* in diabetic TECs (Figure 2H; Figure S2G) and its critical role in interstitial signaling, we next examined the PDGF axis in detail. In healthy testes, TECs serve as the predominant source of PDGFB, establishing paracrine communication with Leydig cells (LCs) and testicular peritubular cells (TPCs) via the PDGFB‐PDGFRB axis (Figure 3A,B; Figure S3B). In diabetes, however, this signaling route was weakened, as evidenced by a significant reduction in the probability of PDGFB‐PDGFRB interactions (Figure 3A,B).

Immunofluorescence staining further corroborated this observation by demonstrating markedly reduced PDGFB expression in diabetic TECs (Figure 3C,D; Figure S3C–E), together with concomitant downregulation of PDGFRB in both LCs and TPCs (Figure 2E–G; Figure S3C). Collectively, these results demonstrate that diabetes compromises TEC‐derived PDGFB production and disrupts receptor availability in target cells, resulting in dysfunction of the PDGF signaling axis at two distinct levels. This systemic impairment highlights severe disruption of TEC‐interstitial cell communication and provides direct molecular evidence for diabetes‐induced dysregulation of the testicular microenvironment.

### 2.4 LC Dysfunction in Diabetes Driven by JUND‐MCL1 Axis Disruption

**FIGURE 4:**
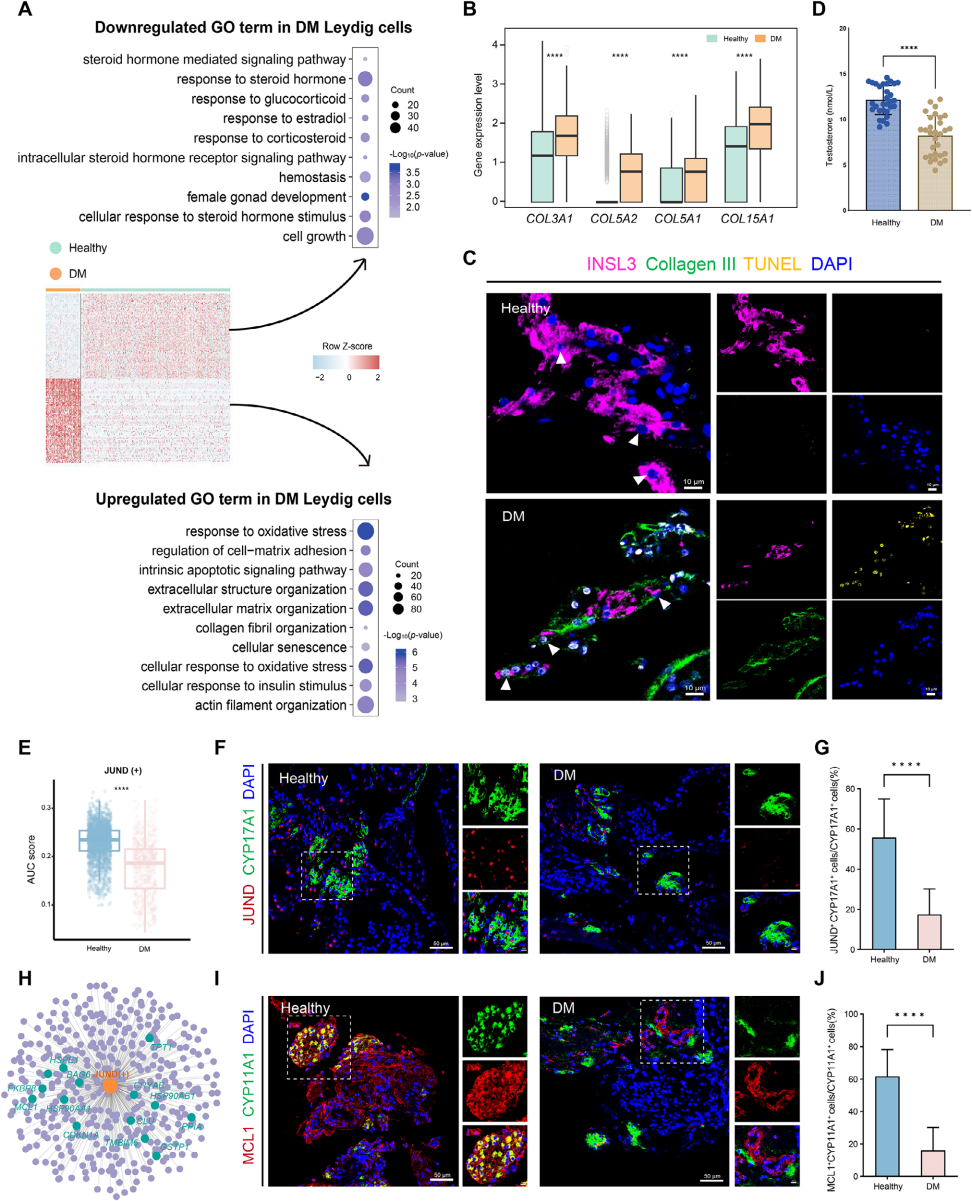
Dysregulated JUND underlies the functional defects of LCs in diabetes. (A) Heatmap showing DEGs in LCs from healthy and DM testes. Rows represent DEGs and columns indicate samples, with expression levels scaled by Z score. Right panel shows GO enrichment analysis of DEGs. Dot size represents the number of genes per GO term, and color intensity indicates statistical significance –Log_10_(*p*‐value). (B) Box plots illustrating the expression of collagen genes in LCs from healthy and DM testes. ****p < 0.0001. (C) Representative immunofluorescence staining of INSL3 (magenta), Collagen III (green), TUNEL (yellow), and DAPI (blue) in testicular sections from healthy and DM patients. Scale bars, 10 µm. (D) Bar plot comparing the testosterone level between healthy individuals (n = 30) and DM patients (n = 30). Statistical significance was determined using the two‐tailed Student's t‐test. ****p < 0.0001. (E) Box plot depicting JUND regulon activity (AUC score) in LCs from healthy and DM testes. (F) Representative immunofluorescence staining of JUND (red) and CYP17A1 (green) in testicular sections from healthy and DM patients. Nuclei are counterstained with DAPI (blue). Insets show magnified views. Scale bars, 50 µm; 10 µm (insets). (G) Quantification of JUND^+^ CYP17A1^+^ LCs among total CYP17A1^+^ cells in healthy and DM testes. Bars show mean ± SD. n = 3 independent biological replicates per group. Statistical significance was determined using the two‐tailed Student's t‐test. ****p < 0.0001. (H) Transcriptional regulatory network derived from JUND in Leydig cells. (I) Representative immunofluorescence staining of MCL1 (red) and CYP11A1 (green) in testicular sections from healthy and DM patients. Nuclei are counterstained with DAPI (blue). Insets show magnified views. Scale bars, 50 µm; 10 µm (insets). (J) Quantification of MCL1^+^ CYP11A1^+^ LCs among total CYP11A1^+^ cells in healthy and DM testes. Bars show mean ± SD. n = 3 independent biological replicates per group. Statistical significance was determined using the two‐tailed Student's t‐test. ****p < 0.0001.

To further characterize how diabetes affects interstitial cell populations, we focused on LCs, a primary downstream target of PDGF signaling, and systematically examined their transcriptional and functional alterations in diabetes. First, we performed pseudotime analysis, which revealed a clear segregation of LCs from healthy and diabetic testes, suggesting diabetes‐induced transcriptional alterations (Figure S4A,B). To gain deeper insight into these changes, we performed GO enrichment analysis, which revealed that diabetic LCs downregulated steroid hormone biosynthesis and endocrine function, while upregulating processes associated with ECM remodeling, oxidative stress responses, apoptosis, and cellular senescence (Figure 4A). Correspondingly, the expression of *COL3A1*, *COL5A2*, *COL5A1*, and *COL15A1* was significantly upregulated in the DM group (Figure 4B), while collagen catabolic processes were suppressed (Figure S4C), indicating ECM accumulation around LCs. This was further supported by Masson's trichrome staining, which revealed interstitial collagen deposition in diabetic testes (Figure S4D). At the cellular level, immunofluorescence further confirmed collagen III deposition around INSL3^+^ LCs (Figure 4C), indicating excessive ECM accumulation that reshapes the pericellular microenvironment into a fibrotic, mechanically stiffened niche.

In parallel, diabetic LCs exhibited stress‐associated transcriptional alterations, characterized by suppressed antioxidant defenses (*SOD1/2/3*, *PRDX1/2*, and *TXN*) (Figure S4E) and a shift toward apoptosis, with increased expression of pro‐apoptotic regulators (*FAF1*, *CRADD*, *GSK3B*, and *BCL2L14*) and reduced expression of anti‐apoptotic genes (*MIF* and *MCL1*) (Figure S4F). Consistent with these transcriptional changes, INSL3^+^ LCs exhibited increased TUNEL positivity in diabetic testes (Figure 4C). Collectively, these findings reveal that diabetic LCs exhibit ECM deposition, cellular stress, and increased apoptosis, which disrupt transcriptional programs essential for LC survival and may impair their steroidogenic function, as evidenced by significantly reduced serum testosterone levels in men with diabetes (Figure 4D).

To uncover upstream regulators underlying LC stress and functional decline in diabetes, we profiled transcription factor regulons and intersected them with DEGs (Figure S4G). JUND stood out, with targets under healthy conditions enriched for anti‐apoptotic and regulation of cell population proliferation programs (Figure S4H) that are essential for maintaining LC viability [26, 27], and thus may support steroidogenic activity. Notably, this protective transcriptional network was markedly weakened in diabetic LCs, as reflected by significant reductions in JUND regulon activity and transcript levels (Figure 4E; Figure S4I), consistent with the enhanced stress and apoptosis in diabetic LCs (Figure 4A–C). Immunofluorescence further confirmed a significant reduction of JUND^+^ CYP17A1^+^ LCs in diabetic testes (Figure 4F,G). Based on regulon analysis, downstream analysis revealed that expression of the anti‐apoptotic factor *MCL1*, a putative downstream target of JUND, was reduced in diabetic LCs (Figure 4H; Figure S4J,K), which was confirmed by immunofluorescence (Figure 4I,J). To functionally validate this regulatory relationship, JUND was silenced in human primary LCs, resulting in a marked reduction in MCL1 expression at both the protein and mRNA levels (Figure S4L–N). Together, these findings define a JUND‐MCL1 transcriptional axis in LCs that is disrupted in diabetes and associated with LC survival.

### 2.5 Diabetes Disrupts the Architecture and Contractile Identity of TPCs

**FIGURE 5:**
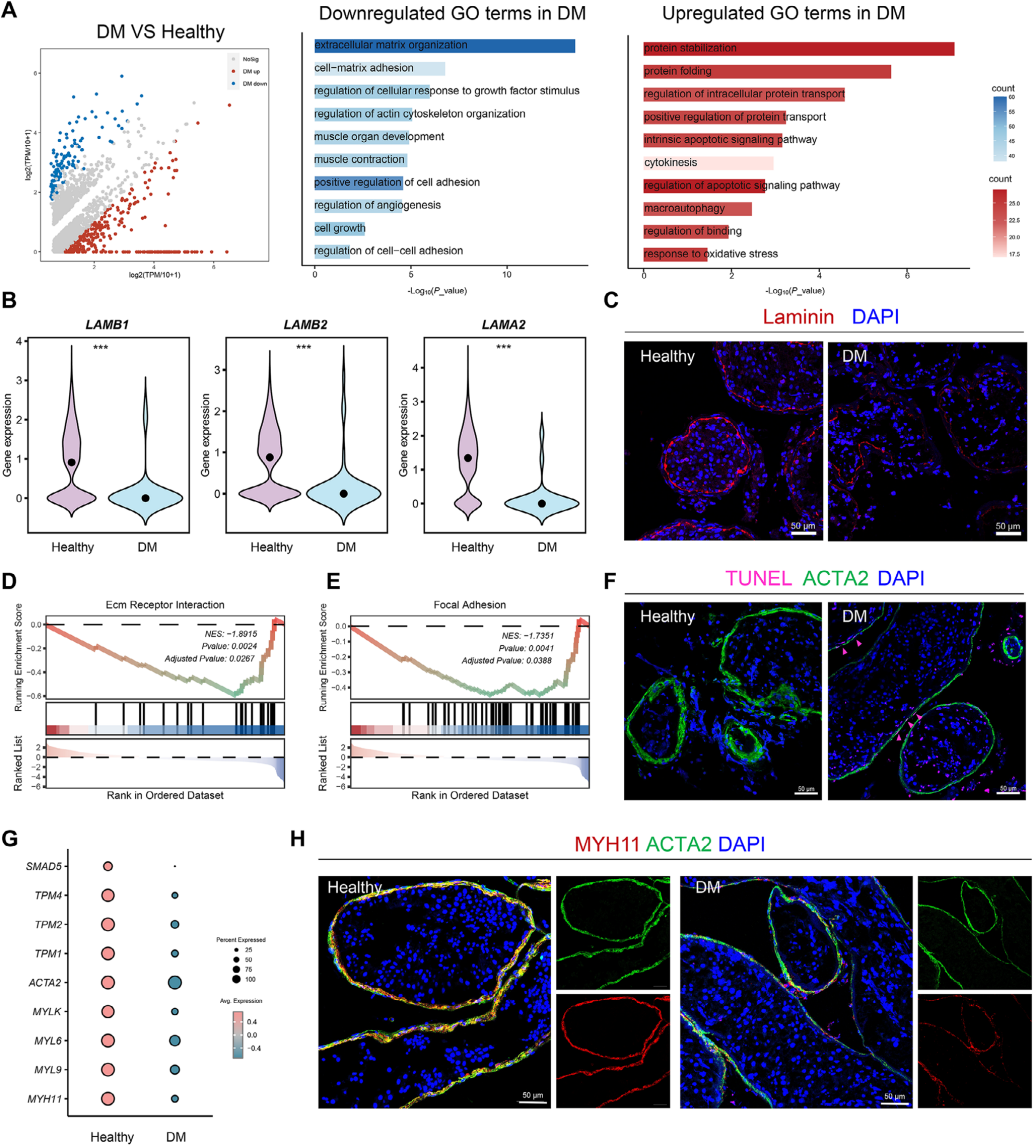
Diabetic TPCs exhibit compromised basement membrane integrity and contractile function. (A) Left: Volcano plot displaying DEGs of TPCs between DM and healthy testes. Right: GO enrichment analysis of downregulated genes (blue) and upregulated genes (red) in TPCs from DM testes compared with healthy controls. Bars indicate –Log_10_(*p*‐value), and bar color reflects gene counts. (B) Violin plots showing the expression of basement membrane genes (*LAMB1, LAMB2, LAMA2*, and *LAMC1*) in testicular peritubular cells (TPCs) from healthy and DM testes. ***p < 0.001. (C) Representative immunofluorescence staining of laminin (red) in testicular sections from healthy and DM patients. Nuclei are counterstained with DAPI (blue). Scale bars, 50 µm. (D‐E) GSEA of functions related to ECM receptor interaction and focal adhesion in TPCs comparing DM and healthy testes. The running enrichment score is shown at the top, and black vertical lines mark the positions of pathway genes in the ranked list. (F) Representative immunofluorescence staining of TUNEL (magenta) and ACTA2 (green) in testicular sections from healthy and DM patients. Nuclei are counterstained with DAPI (blue). Scale bars, 50 µm. (G) Dot plot showing the expression of contractility‐associated genes in TPCs from healthy and DM testes. Dot size represents the proportion of cells expressing each gene, and color intensity indicates the average expression level. (H) Representative immunofluorescence staining of MYH11 (red) and ACTA2 (green) in testicular sections from healthy and DM patients. Nuclei are counterstained with DAPI (blue). Scale bars, 50 µm.

Given that TPCs also engage TEC‐derived PDGF signaling, we next systematically characterized their transcriptional and functional landscape under diabetic conditions. GO enrichment analysis showed that downregulated genes in diabetic TPCs were enriched for ECM organization and cell‐matrix adhesion (Figure 5A), with a reduction of core matrisome components such as *COL1A1*, *COL3A1*, *COL4A1/2*, and multiple laminin subunits (*LAMB1/2* and *LAMA2*) (Figure 5B; Figure S5A). Consistent with these transcriptional alterations, laminin immunofluorescence showed fragmented and irregular basement membrane architecture in diabetic testes (Figure 5C). GSEA further demonstrated significant downregulation of ECM receptor interaction and focal adhesion pathways in diabetic TPCs (Figure 5D,E), underscoring a loss of cell‐matrix connectivity and structural support.

In parallel, diabetic TPCs showed transcriptional signatures of increased apoptotic signaling (Figure 5A), characterized by downregulation of anti‐apoptotic factors (*XIAP*, *MCL1*, and *BCL2*) and upregulation of pro‐apoptotic genes (*FAS* and *CYCS*) (Figure S5B). Consistently, ACTA2^+^ TPCs in diabetes displayed increased TUNEL positivity, corroborating these molecular changes (Figure 5F). Furthermore, contractility‐associated programs were suppressed in diabetic TPCs, as evidenced by downregulation of muscle contraction processes and reduced expression of key contractile regulators (*TPM2*, *ACTA2*, *MYLK*, *MYL9*, and *MYH11*) (Figure 5A–G; Figure S5C,D). Double immunofluorescence confirmed reduced and disorganized MYH11 within ACTA2^+^ TPCs in diabetic testes (Figure 5H), indicating erosion of their smooth muscle‐like contractile phenotype.

Together, these findings reveal that diabetes drives deterioration of TPC structure and function, manifested as disorganization of the ECM and basement membrane, increased apoptosis, and loss of contractile identity. This deterioration compromises the mechanical and structural integrity of the seminiferous tubules, thereby potentially impairing both mechanical transport and germ cell support.

### 2.6 Reactivation of PDGF Signaling Rescues Interstitial Cell Survival and Function in Diabetes

**FIGURE 6:**
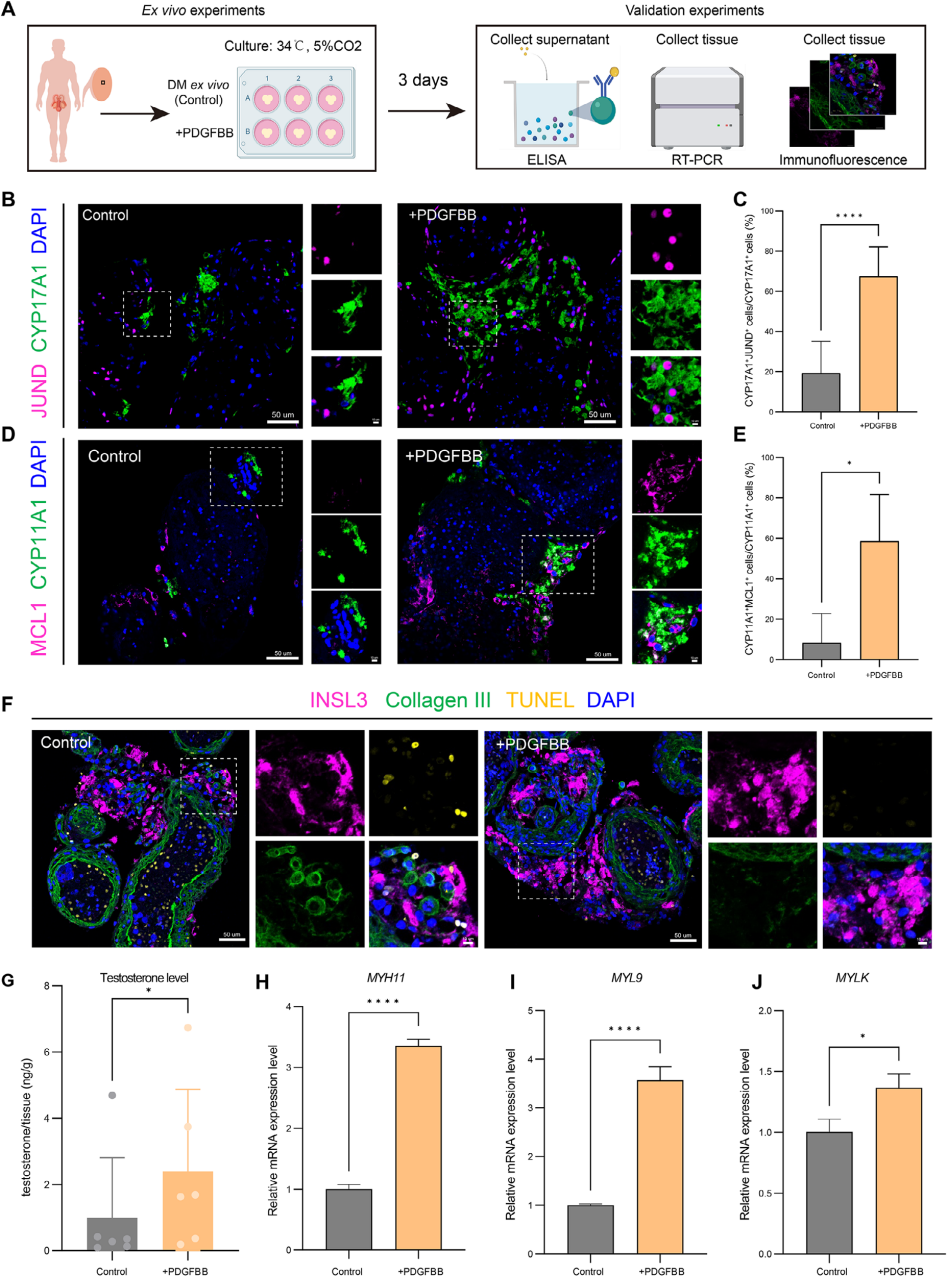
PDGF‐BB supplementation ameliorates interstitial cell dysfunction. (A) Schematic diagram of *ex vivo* culture of human testicular tissue derived from diabetic patients (n = 6). Tissue fragments were cultured with or without PDGF‐BB supplementation (100 ng/mL) for 3 days, followed by ELISA, RT‐PCR, and immunofluorescence validation. This plot was created with BioRender.com. (B) Representative immunofluorescence staining of JUND (magenta) and CYP17A1 (green) in *ex vivo* cultured diabetic human testicular tissue with or without PDGF‐BB treatment (100 ng/mL). Nuclei are counterstained with DAPI (blue). Insets show magnified views. Scale bars, 50 µm; 10 µm (insets). (C) Bar graph quantifying the proportion of JUND^+^ CYP17A1^+^ LCs in *ex vivo* cultured diabetic human testicular tissue with or without PDGF‐BB supplementation (100 ng/mL). Bars represent the mean ± SD. n = 3 independent biological replicates per group. ****p < 0.0001. (D) Representative immunofluorescence staining of MCL1 (magenta) and CYP11A1 (green) in *ex vivo* cultured diabetic human testicular tissue with or without PDGF‐BB treatment (100 ng/mL). Nuclei are counterstained with DAPI (blue). Insets show magnified views. Scale bars, 50 µm; 10 µm (insets). (E) Bar graph quantifying the proportion of MCL1^+^ CYP17A1^+^ LCs in *ex vivo* cultured diabetic human testicular tissue with or without PDGF‐BB supplementation (100 ng/mL). Bars represent the mean ± SD. n = 3 independent biological replicates per group. ****p < 0.0001. (F) Representative immunofluorescence staining of INSL3 (magenta), Collagen III (green), and TUNEL (yellow) in *ex vivo* cultured diabetic human testicular tissue with or without PDGF‐BB (100 ng/mL). Nuclei are counterstained with DAPI (blue). Insets show magnified views. Scale bars, 50 µm; 10 µm (insets). (G) Bar graph showing testosterone concentrations measured by ELISA in supernatants from *ex vivo* cultured diabetic human testicular tissue with or without PDGF‐BB supplementation (100 ng/mL). Bars represent mean ± SD. *p < 0.05. (H‐J) RT‐PCR analysis of *MYH11, MYL9*, and *MYLK* expression in *ex vivo* cultured diabetic human testicular tissue with or without PDGF‐BB supplementation (100 ng/mL). Bars represent mean ± SD. n = 3 independent biological replicates per group. ****p < 0.0001, *p < 0.05. Statistical significance was determined using the two‐tailed Student's t‐test (C, E, G, H, I, J).

Recognizing that disruption of TEC‐derived PDGF signaling accompanies interstitial cell dysfunction in diabetes, we next asked whether restoring PDGF‐BB‐mediated paracrine signaling could ameliorate the observed pathological alterations. To this end, human diabetic testicular tissues were cultured *ex vivo* with recombinant PDGF‐BB (100 ng/mL) for 3 days and assessed for both molecular and functional responses (Figure 6A). At the whole‐tissue level, PDGF‐BB increased the expression of pro‐proliferative and anti‐apoptotic genes (e.g., *RAF1*, *CDK4*, and *BCL2*) (Figure S6A–C), suggesting a global improvement in the stress response.

We next examined its impact on specific interstitial cell populations. In LCs, PDGF‐BB significantly increased the fraction of JUND^+^CYP17A1^+^ cells (Figure 6B,C) and restored the downstream anti‐apoptotic effector MCL1 (Figure 6D,E), indicating reactivation of the JUND‐MCL1 survival network. Consistently, PDGF‐BB increased LC abundance, reduced apoptosis, and elevated testosterone levels (Figure 6F,G; Figure S6D–F), most likely reflecting expansion of the viable steroidogenic LC pool. In addition, PDGF‐BB also acted on TPCs, restoring contractility‐associated gene expression (*MYH11*, *MYL9*, and *MYLK*) (Figure 6H–J), suggesting partial recovery of their smooth muscle‐like phenotype. Finally, immunofluorescence revealed a reduction in pericellular collagen deposition around LCs following PDGF‐BB treatment (Figure 6F; Figure S6D), indicating that PDGF‐BB alleviates the fibrotic interstitial niche.

**FIGURE 7:**
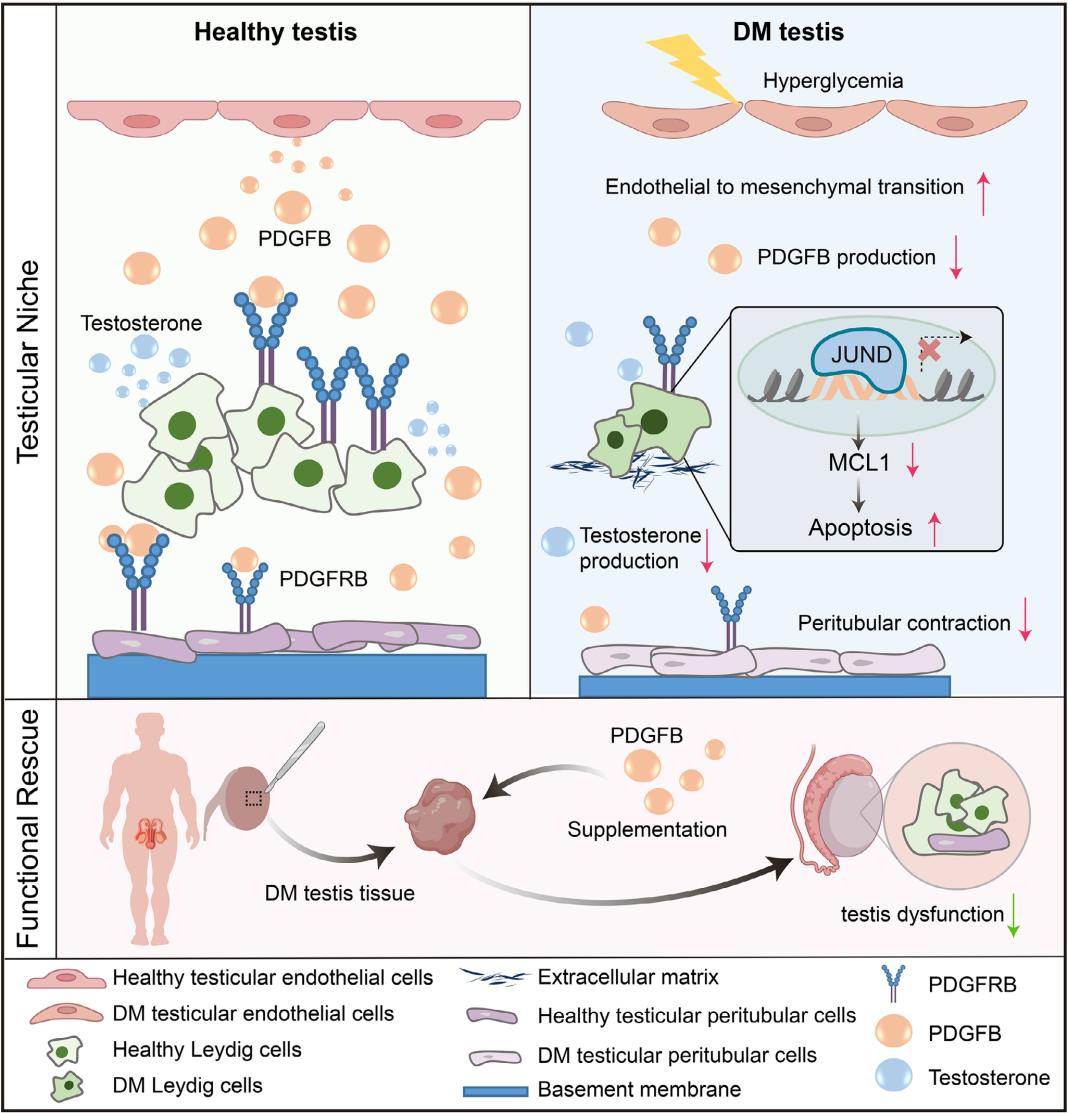
TEC‐derived PDGF signaling disruption impairs interstitial homeostasis and is partially restored by PDGF‐BB supplementation. In healthy testes, TECs secrete PDGFB, which engages PDGFRB on LCs and TPCs to maintain interstitial homeostasis. Under diabetic conditions, TECs undergo EndMT and show a marked reduction in PDGFB expression. Together with decreased PDGFRB levels in LCs and TPCs, this collapse of the ligand‐receptor axis disrupts intercellular communication and contributes to interstitial dysfunction. Specifically, LCs exhibit profound structural and functional alterations, including excessive ECM deposition, increased apoptosis resulting from inactivation of the JUND‐MCL1 survival pathway, and diminished testosterone production. Meanwhile, TPCs display impaired contractility and thinning of the basement membrane, further destabilizing the interstitial niche. Importantly, supplementation with exogenous PDGF‐BB partially restores this paracrine support, enhancing LC survival and steroidogenic activity and improving TPC contractility, thereby alleviating diabetes‐induced testicular dysfunction. This plot was created with BioRender.com.

Together, these findings establish PDGF‐BB supplementation as an effective strategy to alleviate diabetes‐associated interstitial impairment. Diabetes‐driven dysfunction of TEC‐derived PDGF signaling silences the JUND‐MCL1 axis in LCs, promoting apoptosis accompanied by testosterone deficiency and ECM accumulation. Restoring this paracrine pathway with exogenous PDGF‐BB reactivates JUND‐MCL1, protects LCs, partially rescues steroidogenic function, and alleviates ECM deposition (Figure S6G). In parallel, PDGF‐BB also reinstates contractile gene programs in TPCs, highlighting its capacity to restore interstitial homeostasis. Mechanistically, these effects were abolished by CP‐673451 (1 µM), a PDGFRB inhibitor [28], confirming that PDGF‐BB functions via PDGFB‐PDGFRB signaling (Figure S6H–J). Nevertheless, it is worth noting that the functional improvement observed ex vivo remained partial, likely due to inherent limitations of the culture system. Future studies are warranted to validate PDGF‐BB efficacy in vivo and to explore potential combination therapies that could enhance therapeutic outcomes (Figure 7).

## 3 Discussion

Diabetes‐associated male reproductive dysfunction has long been recognized, but effective therapies remain lacking [29, 30]. Current interventions are largely confined to systemic glycemic control and testosterone replacement therapy, both of which provide limited benefit for restoring intrinsic testicular function [31]. Here, we demonstrate that disruption of the PDGF signaling axis is a primary mechanism of interstitial pathology in diabetic testes. Importantly, exogenous PDGF‐BB supplementation reactivated survival pathways, improved Leydig cell steroidogenesis, and restored peritubular contractility, offering mechanistic insight and translational potential (Figure 7).

Our study identifies TECs as a major paracrine hub that orchestrates interstitial signaling in the human testis. In diabetes, TECs adopt an endothelial‐to‐mesenchymal transition phenotype [32, 33, 34] and concomitantly exhibit a pronounced reduction in PDGFB secretion. This coordinated disruption of cellular morphological and paracrine signaling capacity renders TECs unable to sustain interstitial homeostasis. However, the upstream origin of this injury remains unclear, whether it is driven directly by hyperglycemia and oxidative stress or secondarily through inflammatory and metabolic disturbances. Future studies should clarify this question, which will be an important step for understanding how systemic metabolic stress is transduced into the local testicular environment.

An important finding of this study is that diabetes disrupts the PDGF paracrine axis, thereby destabilizing interstitial homeostasis. Specifically, LCs exhibit increased apoptosis and impaired steroidogenesis, while TPCs show diminished contractile and structural support. These findings position PDGF signaling as a unifying upstream driver, reframing diabetic testicular pathology from fragmented observations of individual cell types into an integrated framework, and offering new insights into the mechanisms of male reproductive dysfunction in metabolic disease. However, it should be noted that the impact of PDGF signaling is highly dependent on cell type, acting either as a reparative factor [35, 36] or as a pathological driver [37]. In diabetes, this duality becomes evident, as sustained overactivation drives fibrotic remodeling [38, 39, 40], whereas insufficient PDGF‐BB creates a repair bottleneck that can be relieved by targeted supplementation, restoring angiogenesis and regeneration [41, 42, 43, 44]. Intriguingly, the pathological pattern in diabetic testes exemplifies a signal‐deficient paradigm. Exogenous PDGF‐BB supplementation alleviates apoptosis in LCs and restores both testosterone levels and peritubular contractility. Mechanistically, PDGF‐BB treatment was accompanied by increased JUND activity and upregulation of its downstream anti‐apoptotic effector MCL1 in LCs, suggesting the presence of a PDGF‐JUND‐MCL1 axis that may provide protection against apoptosis in interstitial cells [26, 45, 46]. Although further validation is warranted, this axis provides a plausible rationale for the protective effects of PDGF‐BB.

Finally, we considered the translational implications of our findings for male reproductive health. Notably, PDGF‐BB has already been approved for the treatment of diabetic foot ulcers [47]. and has been incorporated into periodontal and bone regeneration therapies [48, 49, 50], providing a strong precedent for translational repurposing. In the testis, supplementation could go beyond current management by directly repairing the disrupted interstitial microenvironment, thereby offering a more causative therapeutic approach. Moreover, the concomitant downregulation of *PDGFB* and *PDGFRB* may represent candidate biomarkers for identifying patients most likely to benefit from such interventions. Nevertheless, a substantial gap remains between our ex vivo observations and clinical translation. A major challenge lies in achieving safe, effective, and spatially controllable delivery of PDGF‐BB to the testis. Moreover, ex vivo models cannot fully recapitulate the systemic physiological complexity present in vivo, which may result in divergent therapeutic effects in living organisms. Accordingly, comprehensive in vivo studies will be essential to evaluate the pharmacokinetics, biodistribution, durability, and safety of PDGF‐BB‐based interventions. Importantly, given the multifactorial nature of diabetic testicular injury, effective restoration of testicular homeostasis will likely require modulation of PDGF signaling in combination with additional therapeutic strategies.

This study has several limitations. Although single‐cell RNA sequencing enables high‐resolution profiling, technical artifacts associated with tissue dissociation and limited sensitivity for transcript detection remain inherent limitations. Furthermore, diabetes is a heterogeneous disease with variability in duration, glycemic control, and comorbidities, and larger patient cohorts will be needed to confirm the robustness of our findings. Despite these limitations, our findings provide a conceptual advance by positioning TEC‐derived PDGF signaling as a central regulator of interstitial homeostasis and a promising therapeutic target for restoring testicular function in diabetes.

## 4 Method Details

### 4.1 Ethics Statement

Adult testicular samples for single‐cell RNA sequencing were obtained from three obstructive azoospermia patients diagnosed with type 2 diabetes. Additional testicular samples for histological validation and *ex vivo* tissue culture were collected from 30 obstructive azoospermia patients with type 2 diabetes and 30 with normal spermatogenesis. All samples were obtained during surgical testicular sperm extraction. Serum testosterone levels were measured in all donors. Detailed clinical characteristics and statistical comparisons between healthy and diabetic groups are presented in Table S1. All tissue and serum samples were collected with informed consent prior to clinical procedures. The study was approved by the Institutional Review Board of Peking University Third Hospital (IRB00006761‐M2022692) and conducted in accordance with the principles of the Declaration of Helsinki.

### 4.2 Testis Sample Collection

Human testicular samples were obtained from patients undergoing microdissection testicular sperm extraction. Immediately after surgical removal, fresh tissue fragments were placed on ice in pre‐cooled sterile saline. Following thorough PBS washing, the testicular tissues were carefully cut into approximately 1 × 1 × 1 cm blocks for subsequent experiments, ensuring consistency and preservation of tissue integrity.

### 4.3 Sample Fixation for Histological Staining

Testicular tissues were fixed in 4% paraformaldehyde (PFA) for 24 h at room temperature and subsequently rinsed three times with 0.01 M PBS (10 min each) to remove residual fixative. The fixed samples were then dehydrated through a graded ethanol series, with sequential immersion in 50%, 75%, 80%, 95% (twice), and 100% (twice) ethanol. Each step lasted 30–50 min, with the duration adjusted according to ambient temperature. Following dehydration, tissues were transferred into a mixed solution of ethanol and xylene (1:1) and then placed sequentially into xylene I and xylene II to ensure complete clearing. Finally, the samples were embedded in paraffin for long‐term storage and subsequent sectioning.

### 4.4 Masson‐trichrome Staining

Paraffin‐embedded tissue sections (5 µm thick) were deparaffinized in xylene, rehydrated through a graded ethanol series, and rinsed in distilled water. Sections were incubated in potassium dichromate solution at room temperature overnight, washed in running tap water, and stained with freshly prepared Weigert's iron hematoxylin for 1 min. After washing in tap water, nuclear differentiation was performed using 1% acid alcohol for 1 min, followed by another rinse in tap water. Sections were stained with Biebrich scarlet‐acid fuchsin solution for 10 min, differentiated in phosphomolybdic acid for 5 s, and stained in aniline blue for 10 s. Slides were then treated sequentially in two changes of 1% acetic acid for 5 s each. Finally, sections were dehydrated in ethanol, cleared in xylene, and mounted with a resinous medium.

### 4.5 Cell Culture and Transfection

Primary human Leydig cells were purchased from ScienCell Research Laboratories (Carlsbad, CA, USA; Catalog #4510) and cultured in Leydig Cell Medium (ScienCell, Cat. #4511) at 37°C in a humidified atmosphere containing 5% CO_2_, as previously described [51]. Small interfering RNAs (siRNAs) targeting human JUND were synthesized by Tsingke Biotechnology Co., Ltd. (Beijing, China). The siRNA sequences were as follows: siJUND‐sense, 5′‐GCCUCAUCAUCCAGUCCAA‐3′; siJUND‐antisense, 5′‐UUGGACUGGAUGAUGAGGC‐3’. For siRNA transfection, Lipofectamine RNAiMAX Reagent (Invitrogen, Cat. #13778150) was used according to the manufacturer's instructions (Invitrogen, Carlsbad, CA, USA).

### 4.6 Immunofluorescence Staining

The paraffin‐embedded tissue sections were deparaffinized sequentially in xylene I, xylene II, a 1:1 mixture of xylene and ethanol, 100% ethanol (twice), 95% ethanol, 85% ethanol, 70% ethanol, and 50% ethanol (20 min each), followed by PBS washing for 5 min on a shaker. Antigen retrieval was performed in ∼300 mL Tris‐EDTA buffer (pH 9.0, 0.05% Tween 20) using a microwave oven, with high power for 6 min until boiling, followed by two cycles of low power heating (20 min) with brief cooling intervals. After cooling to room temperature, tissue boundaries were circled with a hydrophobic pen, and sections were blocked with 5% BSA/PBS for 30 min in a humidified chamber. Sections were incubated overnight at 4°C with primary antibodies diluted in blocking buffer: anti‐ACTA2 (ab7817, Abcam, 1:500), anti‐CD31 (11265‐1‐AP, Proteintech, 1:200), anti‐PDGFB (ab23914, Abcam, 1:300), anti‐INSL3 (NBP1‐81223, Novus Biologicals, 1:200), anti‐PDGFRB (3169s, Cell Signaling Technology, 1:200), anti‐collagen III (ab23445, Abcam, 1:100), anti‐JUND (ab181615, Abcam, 1:300), anti‐CYP17A1 (94004s, Cell Signaling Technology, 1:200), anti‐MCL1 (15825‐1‐AP, Proteintech, 1:500), anti‐CYP11A1 (ab272494, Abcam, 1:200), anti‐Laminin (ab11575, Abcam, 1:100), and anti‐MYH11 (ab133567, Abcam, 1:500). The next day, slides were rewarmed for 30 min and washed with PBST (1 × PBS with 0.1% Tween 20, 3 × 10 min). Sections were then incubated with secondary antibodies diluted in PBST for 1 h at room temperature in the dark, followed by PBST washes (3 × 5 min). Finally, 10 µL of antifade mounting medium containing DAPI was applied to each section, coverslips were mounted, and the edges were sealed with nail polish before imaging.

### 4.7 TUNEL Assay

Apoptotic cells in paraffin‐embedded testicular tissue sections were detected using the in situ cell death detection kit (Roche, 11684817910). Fluorescein labeling was performed according to the manufacturer's instructions. Briefly, tissue sections were deparaffinized in xylene, rehydrated through a graded ethanol series, and permeabilized with 0.1% Triton X‐100 in 0.1% sodium citrate buffer for 15 min at room temperature. After PBS washing, sections were incubated with the TUNEL reaction mixture in a humidified chamber at 37°C for 1 h. Nuclei were counterstained with DAPI, and images were acquired using a fluorescence microscope.

### 4.8 Human Blood Sample Collection and Testosterone Measurement

Blood samples from patients with DM (n = 30) who were admitted to Peking University Third Hospital for testicular sperm aspiration were collected with informed consent (IRB00006761‐M2022692). Meanwhile, 30 blood samples from healthy males without DM were collected as controls. Serum testosterone was measured through an automated chemiluminescent immunoassay (Siemens IMMULITE 2000 immunoassay system; Siemens Healthcare Diagnostics, Shanghai, China).

### 4.9 Ex Vivo Culture and PDGF‐BB Supplementation

Short‐term *ex vivo* culture of human testicular tissue was adapted from previous protocols with minor modifications [52]. Briefly, cryopreserved testicular tissues obtained from diabetic patients were rapidly thawed, washed twice with PBS, and cut into small fragments (∼2–4 mm^2^). For statistical consistency, tissue pieces were weighed and placed in 2 mL of culture medium per well of a 6‐well plate. The culture medium consisted of KO‐DMEM (Invitrogen, 10829018) supplemented with 10% knock‐out serum replacement (Invitrogen, 10828028) and 1% penicillin/streptomycin (Invitrogen, 15140122). Cultures were maintained at 34°C in a humidified incubator with 5%CO_2_ for 3 days, and the medium was changed every other day. Where indicated, recombinant human PDGF‐BB (R&D Systems, 220‐BB‐010) was added at a final concentration of 100 ng/mL. In parallel, cryopreserved testicular biopsy tissues from healthy donors were cultured under identical conditions and treated with vehicle control, PDGF‐BB (100 ng/mL), or PDGF‐BB (100 ng/mL) plus the PDGFRB inhibitor CP‐673451 (1 µm, MedChemExpress, HY‐12050).

### 4.10 Testosterone Measurement by ELISA

At the end of *ex vivo* culture, supernatants were collected from each well and centrifuged at 500 × g for 5 min at 4°C to remove debris. Testosterone levels in culture supernatants from *ex vivo* cultured testicular tissue were determined using a testosterone ELISA kit (Solarbio, SEKSM‐0003) following the manufacturer's instructions. Briefly, standards and appropriately diluted samples (100 µL each) were added to individual wells of a 96‐well plate, sealed with an adhesive strip, and incubated at 37°C for 2 h. After discarding the liquid, 100 µL of biotin‐conjugated antibody (1×) was added and incubated for 1 h at 37°C. Wells were washed three times with wash buffer, followed by the addition of 100 µL of HRP‐conjugated avidin (1×) and incubation for another 1 h at 37°C. After five additional washes, 90 µL of TMB substrate was added, and the plate was incubated in the dark at 37°C for 15–30 min. The reaction was terminated by adding 50 µL of stop solution, and absorbance was measured at 450 nm within 5 min using a microplate reader.

### 4.11 Western Blot

Human primary Leydig cells were lysed in RIPA buffer (50 mmol/L Tris‐HCl, pH 7.4, 150 mmol/L NaCl, 1% NP‐40, 1% sodium deoxycholate, and 0.1% SDS) supplemented with a protease inhibitor cocktail. Protein concentrations were determined using the Pierce BCA Protein Assay Kit (Thermo Fisher Scientific, Cat. #23225). Equal amounts of protein were mixed with SDS loading buffer and boiled at 95°C for 10 min. Proteins were separated by SDS‐PAGE and electrotransferred onto PVDF membranes (Millipore). Membranes were blocked with 5% non‐fat milk and incubated with primary antibodies overnight at 4°C. After washing, membranes were incubated with HRP‐conjugated secondary antibodies. Protein bands were visualized using a chemiluminescence imaging system (ChemiScope 6100, China). The following antibodies were used: anti‐JUND (Abcam, ab181615, 1:1,000), anti‐MCL1 (Proteintech, 16225‐1‐AP, 1:1,000), anti‐GAPDH (Cell Signaling Technology, 5174S, 1:1,000), and goat anti‐rabbit IgG (Beyotime, A0208, 1:1,000).

### 4.12 RT‐PCR Analysis of Ex Vivo Cultured Testicular Tissue

Total RNA was isolated from *ex vivo* cultured human testicular tissue fragments (with or without PDGF‐BB treatment) and from human primary LCs following siRNA transfection using TRIzol reagent (Invitrogen, 15596026) according to the manufacturer's instructions. RNA (1 µg) was reverse transcribed to generate cDNA. Quantitative PCR was performed on a real‐time PCR system following the cycling conditions recommended by the manufacturer, including melt curve analysis to confirm single products. Gene expression was normalized to GAPDH as the internal reference, and relative expression was calculated using the 2^−ΔΔCt method. Data are presented as mean ± SD from three independent biological replicates. Primer sequences used for target gene amplification were as follows:

*RAF1*:F: Atgcgtcgtatgcgagagtctgt, R: Aaggtgaaggcgtgaggtgtaga. *BCL2*: F: Ttcgccgagatgtccagcca, R: Gcatcccagcctccgttatcct. *CDK4*: F: Aaattggtgtcggtgcctatggg, R: Aagcctccagtcgcctcagtaa. *MYH11*: F: Tgctcaatgcctcctccgacaa, R: Gtgttgcgtagcgtggtcatca. *MYL9*: F: Aagccaagaccaccaagaagcg, R: Aggcgttgcgaatcacatcctc. *Mylk*: F: Gagaacagcgagaatggcagcaa, R:Tccgaatgtcagaggcacaaggt. *MCL1*: F: Tttcagcgacggcgtaacaaact, R: Cagcacattcctgatgccacctt

### 4.13 Sample Preparation for Single‐Cell RNA Sequencing

For each experiment, a cryovial of testicular tissue was rapidly thawed and rinsed twice with PBS. Tissue fragments were mechanically dissociated with sterile razor blades, followed by enzymatic digestion with 1 mg/mL collagenase type IV (Solarbio, C8160) and 1 mg/mL DNase I (Solarbio, D8071). The suspension was then further digested with trypsin‐EDTA (Solarbio, T1300) and 1 mg/mL DNase I at 37°C for 5 min. Single‐cell suspensions were filtered through 70 µm strainers (Miltenyi, 130‐098‐462) and washed with DPBS (Gibco, 14040133). Finally, cells were resuspended in DPBS containing 0.4% BSA (Sigma, B2064) at a concentration of ∼1,000 cells/µL, yielding a single‐cell suspension suitable for downstream single‐cell RNA sequencing.

### 4.14 Single‐Cell RNA‐seq Library Preparation and Sequencing

Single‐cell RNA‐seq libraries were prepared following the manufacturer's instructions for the Chromium Next GEM Single Cell 3ʹ Reagent Kits v3.1 (10x Genomics). Briefly, single‐cell suspensions were diluted to a target capture of ∼5,000 cells per channel and loaded together with the master mix onto a Chromium Next GEM Chip G to generate Gel Bead‐in‐Emulsions (GEMs). After reverse transcription and post‐GEM cleanup, cDNA was amplified for 12 cycles. Sequencing libraries were constructed following the 10x Genomics protocol and sequenced on an Illumina NovaSeq 6000 platform (Illumina, San Diego, USA) by Annoroad Gene Technology Co., Ltd. (Beijing, China). The read configuration was as follows: 28 cycles for Read 1, 10 cycles for i5 index, 10 cycles for i7 index, and 90 cycles for Read 2.

### 4.15 Processing of Single‐Cell RNA‐seq Data

Raw sequencing data were demultiplexed using the *mkfastq* function in Cell Ranger v7.0.0. FASTQ files were processed with the *count* function under default settings, including alignment to the *GRCh38* human reference genome (STAR aligner), filtering, and UMI counting. The resulting UMI count matrices from five samples were loaded into R (v4.3.2) using the *Read10X* function in *Seurat* [53]. v4.3.2 (https://satijalab.org/seurat/index.html, R package, v4.3.2). Sample identifiers were appended to cell barcodes, and the datasets were merged into a single Seurat object using the *merge* function. Cells were retained if they expressed >1 000 and <10 000 genes and had <50% mitochondrial read content. After quality control, healthy samples and diabetes samples were integrated using the Harmony package [54]. (v1.1.0), yielding a combined dataset. Dimensionality reduction was performed using the top 25 principal components (PCs) derived from the top 5 000 highly variable genes, followed by UMAP visualization and clustering. Cell types were annotated based on canonical marker expression, following Seurat's standard workflow. For subpopulation analysis, the subset function was used to extract specific cell types and construct new Seurat objects.

### 4.16 Cell–Cell Communication Analysis

Cell‐cell communication was analyzed using CellChat [55]. (https://github.com/sqjin/CellChat, R package, v1.4.0). Briefly, single‐cell transcriptomic profiles and metadata were imported to initialize a CellChat object through the *createCellChat* function. Ligand‐receptor interactions were inferred using the “Secreted Signaling” database provided within the package. Following the developer's workflow, gene expression data were projected onto a protein‐protein interaction (PPI) network to contextualize potential signaling events. Communication probabilities between cell groups were estimated with the *computeCommunProb* function, and pathway‐level signaling interactions were subsequently evaluated using the *computeCommunProbPathway* function. To quantify the contribution of different cell types to intercellular communication, outgoing signaling strength was calculated across all major testicular cell populations. We further compared the outgoing signaling strength of TECs across different pathological conditions. Finally, we set TECs as *the source, use*, and other cell types as the *target.use* to delineate TECs derived signaling pathways and their alterations under diabetic conditions.

### 4.17 Differentially Expressed Genes and Functional Enrichment Analysis

Differentially expressed genes (DEGs) were identified using the *FindMarkers* function in Seurat, with thresholds set at avg_Log_2_FC > 0.25 and adjusted *p* < 0.05. Gene Ontology (GO) enrichment analysis was performed for the Biological Process (BP) category using the R package clusterProfiler [56]. (v4.6.2). To quantify functional activity at the single‐cell level, the *AddModuleScore* function in Seurat was applied, enabling calculation of average expression scores for gene sets enriched in specific biological processes across different conditions. Visualization of enrichment and scoring results was performed using ggplot2 (v3.4.4).

### 4.18 Gene Set Enrichment Analysis

Gene set enrichment analysis (GSEA) was performed using the clusterProfiler R package (v4.8.3) to identify pathways enriched between healthy and DM groups. DEGs were ranked by log2 fold change, and enrichment was tested against the MSigDB C5 gene sets using default parameters. Normalized enrichment scores (NES), nominal *p*‐values, and adjusted *p*‐values (Benjamini‐Hochberg correction) were calculated following the standard algorithm. Enrichment plots were generated with the GseaVis R package (v0.1.1) and further customized in ggplot2 (v3.4.4).

### 4.19 Principal Component Analysis of TECs Subclusters

To assess transcriptional divergence among TECs subclusters, we performed principal component analysis (PCA). Briefly, variable features were identified from the TECs dataset, and average expression values for each subcluster (TEC1‐TEC4) were computed with the *AverageExpression* function in Seurat (v4.3.2), yielding a gene‐by‐subcluster expression matrix. PCA was performed on the transposed matrix using the *prcomp* function in the stats package (v4.2.3). Finally, we used the ggplot2 package (v3.4.4) to generate plots in which distinct colors and shapes denoted different TECs subclusters.

### 4.20 Scenic Analysis of LCs

To investigate transcriptional regulators underlying LCs reprogramming, we applied single‐cell regulatory network inference and clustering (SCENIC) using pySCENIC [57]. (v0.12.1). The human cisTarget motif and ranking databases (hg38) were retrieved from the Aerts Lab resource (https://resources.aertslab.org/cistarget). The analysis was performed in three steps. First, co‐expression modules were inferred from the gene expression matrix using the ‘pyscenic grn’ function. Next, candidate TF‐target interactions were refined through motif enrichment analysis with the ‘pyscenic ctx’ function. Finally, regulon activity scores (RAS) were calculated at the single‐cell level using the ‘pyscenic aucell’ function. Regulon specificity scores (RSS) were then calculated to compare transcription factor activity between healthy and DM LCs. Key regulons were prioritized based on differential RSS and target enrichment. The resulting gene regulatory networks were exported and visualized in Cytoscape (https://cytoscape.org) to illustrate TF‐target interactions.

### 4.21 Pseudotime Trajectory Analysis of LCs

Pseudotime trajectory analysis of LCs was performed using Monocle2 [58]. (v2.22.0). Expression data from LCs were extracted from the Seurat object and converted into a Monocle CellDataSet object. Dimensionality reduction was conducted using the DDRTree algorithm implemented in the *reduceDimension* function, and trajectories were constructed with the *orderCells* function following the default pipeline. No predefined biological root state was imposed. Instead, Monocle was allowed to infer the trajectory origin algorithmically based on the DDRTree manifold topology, and pseudotime values were interpreted as relative ordering of cell states. Cells were ordered along pseudotime to infer dynamic state transitions, and differences between healthy and DM conditions were visualized by overlaying metadata onto the trajectory plots. All visualizations were generated using Monocle2 and customized in ggplot2 (v3.4.4).

### 4.22 Statistical Analysis

Data were shown as the mean ± SD, as indicated in the figure legends. All statistical analyses were performed using R Statistical Software (v4.4.2). Data normality was assessed using the Shapiro‐Wilk test. For comparisons between two groups, a two‐tailed unpaired Student's t‐test was applied to normally distributed data, whereas the Wilcoxon rank‐sum test was used for non‐normally distributed data. For comparisons involving more than two groups, statistical significance was determined using one‐way analysis of variance (ANOVA) followed by Tukey's post hoc test, or using the Kruskal‐Wallis test when data did not meet normality assumptions. When the Kruskal‐Wallis test was significant, pairwise comparisons were performed using the Wilcoxon rank‐sum test with Benjamini‐Hochberg correction for multiple comparisons. Significance levels are indicated as follows: ^*^ *p* < 0.05; ^**^ *p* < 0.01; ^***^ *p* < 0.001; ^****^ *p* < 0.0001.

## Author Contributions

X.W., Z.Z., J.G., and Q.L. conceived the study. H.J. oversaw the overall study. W.Z. and Y.T. performed computational data analysis. L.C., X.L., Q.J., and Z.Z. assisted with data interpretation. K.H., J.C., and Y.F. performed scRNA‐seq library construction and sequencing. W.Z. and S.G. performed histological experiments and confocal photography. W.Z., K.H., and X.W. wrote the manuscript with input from all authors.

## Conflicts of Interest

All authors state that there is no conflict of interest.

## Supporting information




**Supporting File 1**: advs74233‐sup‐0001‐SuppMat.docx.

**Supporting File 2**: advs74233‐sup‐0002.FigS1.pdf.

**Supporting File 3**: advs74233‐sup‐0003.FigS2.pdf.

**Supporting File 4**: advs74233‐sup‐0004.FigS3.pdf.

**Supporting File 5**: advs74233‐sup‐0005.FigS4.pdf.

**Supporting File 6**: advs74233‐sup‐0006.FigS5.pdf.

**Supporting File 7**: advs74233‐sup‐0007.FigS6.pdf.

**Supporting File 8**: advs74233‐sup‐0008‐TableS1.xlsx.

## Data Availability

The raw sequencing data reported in this paper have been deposited in the Genome Sequence Archive at the National Genomics Data Center, China National Center for Bioinformation (Beijing Institute of Genomics, Chinese Academy of Sciences), and are publicly accessible at https://ngdc.cncb.ac.cn/gsa‐human [59, 60]. under accession number GSA‐Human: HRA010789. The published data of healthy testicular single‐cell transcriptomic datasets [61, 62]. were downloaded from GEO under accession numbers GSE106487 and GSE182786. In addition, we integrated previously published data from diabetic patients’ testes (GSA‐Human: HRA000976) [9]. The scRNA‐seq datasets of patients with KS [63, 64, 65]. (GSE130151, GSE149512 and GSE169062), AZFa deletion [64]. (GSE149512), and NOA [64, 65, 66]. (GSE154535, GSE149512 and GSE169062) were all obtained from GEO. The data of CR patients [67] were accessed via the Genome Sequence Archive under accession number GSA‐Human: HRA006558. All the mentioned data were downloaded and analyzed using Seurat with batch correction by Harmony.

## References

[1] J. Guo , E. J. Grow , H. Mlcochova , et al., “The Adult Human Testis Transcriptional Cell Atlas,” Cell Research 28 (2018): 1141–1157. 30315278 10.1038/s41422-018-0099-2 PMC6274646

[2] J. Kalucka , L. P. M. H. De Rooij , J. Goveia , et al., “Single‐Cell Transcriptome Atlas of Murine Endothelial Cells,” Cell 180 (2020): 764–779. 32059779 10.1016/j.cell.2020.01.015

[3] H. G. Augustin and G. Y. Koh , “A Systems View of the Vascular Endothelium in Health and Disease,” Cell 187 (2024): 4833–4858. 39241746 10.1016/j.cell.2024.07.012

[4] W. C. Aird , “Endothelial Cell Heterogeneity,” Cold Spring Harbor Perspectives in Medicine 2 (2012): a006429–a006429. 22315715 10.1101/cshperspect.a006429 PMC3253027

[5] D. H. Bhang , B.‐J. Kim , B. G. Kim , et al., “Testicular Endothelial Cells Are a Critical Population in the Germline Stem Cell Niche,” Nature Communications 9 (2018): 4379. 10.1038/s41467-018-06881-z PMC6197186 30348976

[6] W. B. Horton and E. J. Barrett , “Microvascular Dysfunction in Diabetes Mellitus and Cardiometabolic Disease,” Endocrine Reviews 42 (2021): 29–55. 33125468 10.1210/endrev/bnaa025 PMC7846151

[7] L. Long , H. Qiu , B. Cai , et al., “Hyperglycemia Induced Testicular Damage in Type 2 Diabetes Mellitus Rats Exhibiting Microcirculation Impairments Associated with Vascular Endothelial Growth Factor Decreased via PI3K/Akt Pathway,” Oncotarget 9 (2018): 5321–5336. 29435181 10.18632/oncotarget.23915 PMC5797052

[8] D. F. Cameron , F. T. Murray , and D. D. Drylie , “Interstitial Compartment Pathology and Spermatogenic Disruption in Testes from Impotent Diabetic Men,” The Anatomical Record 213 (1985): 53–62. 4073561 10.1002/ar.1092130108

[9] K. Song , X. Yang , G. An , et al., “Targeting APLN/APJ Restores Blood‐testis Barrier and Improves Spermatogenesis in Murine and Human Diabetic Models,” Nature Communications 13 (2022): 7335. 10.1038/s41467-022-34990-3 PMC9705293 36443325

[10] D. Kapoor , H. Aldred , S. Clark , K. S. Channer , and T. H. Jones , “Clinical and Biochemical Assessment of Hypogonadism in Men With Type 2 Diabetes,” Diabetes Care 30 (2007): 911–917. 17392552 10.2337/dc06-1426

[11] M. Grossmann , M. C. Thomas , S. Panagiotopoulos , et al., “Low Testosterone Levels Are Common and Associated with Insulin Resistance in Men with Diabetes,” The Journal of Clinical Endocrinology & Metabolism 93 (2008): 1834–1840. 18319314 10.1210/jc.2007-2177

[12] G. Colleluori , L. Aguirre , R. Dorin , et al., “Hypogonadal Men with Type 2 Diabetes Mellitus Have Smaller Bone Size and Lower Bone Turnover,” Bone 99 (2017): 14–19. 28323146 10.1016/j.bone.2017.03.039 PMC8312374

[13] E. J. Giltay , R. C. Van Der Mast , E. Lauwen , A. C. Heijboer , M. W. M. De Waal , and H. C. Comijs , “Plasma Testosterone and the Course of Major Depressive Disorder in Older Men and Women,” The American Journal of Geriatric Psychiatry 25 (2017): 425–437. 28132748 10.1016/j.jagp.2016.12.014

[14] F. Sesti , R. Pofi , M. Minnetti , M. Tenuta , D. Gianfrilli , and A. M. Isidori , “Late‐Onset Hypogonadism: Reductio ad Absurdum of the Cardiovascular Risk‐Benefit of Testosterone Replacement Therapy,” Andrology 8 (2020): 1614–1627. 32737921 10.1111/andr.12876

[15] P. Wang , S. Zhang , S. Lin , and Z. Lv , “Melatonin Ameliorates Diabetic Hyperglycaemia‐induced Impairment of Leydig Cell Steroidogenic Function through Activation of SIRT1 Pathway,” Reproductive Biology and Endocrinology 20 (2022): 117. 35962432 10.1186/s12958-022-00991-6 PMC9373359

[16] L. Hu , S. Wei , Y. Wu , S. Li , P. Zhu , and X. Wang , “MicroRNA Regulation of the Proliferation and Apoptosis of Leydig Cells in Diabetes,” Molecular Medicine 27 (2021): 104. 34496750 10.1186/s10020-021-00370-8 PMC8425090

[17] A. E. Alsemeh , M. A. Samak , and S. S. A. El‐Fatah , “Therapeutic Prospects of Hydroxytyrosol on Experimentally Induced Diabetic Testicular Damage: Potential Interplay with AMPK Expression,” Cell and Tissue Research 380 (2020): 173–189. 31838605 10.1007/s00441-019-03143-2

[18] A. Thiriot , C. Perdomo , G. Cheng , et al., “Differential DARC/ACKR1 Expression Distinguishes Venular from Non‐venular Endothelial Cells in Murine Tissues,” BMC Biology 15 (2017): 45. 28526034 10.1186/s12915-017-0381-7 PMC5438556

[19] M. Pruenster , L. Mudde , P. Bombosi , et al., “The Duffy Antigen Receptor for Chemokines Transports Chemokines and Supports Their Promigratory Activity,” Nature Immunology 10 (2009): 101–108. 19060902 10.1038/ni.1675 PMC3205989

[20] S. Cambier , M. Gouwy , and P. Proost , “The Chemokines CXCL8 and CXCL12: Molecular and Functional Properties, Role in Disease and Efforts towards Pharmacological Intervention,” Cellular & Molecular Immunology 20 (2023): 217–251. 36725964 10.1038/s41423-023-00974-6 PMC9890491

[21] F. Liu , H. Fu , Z. Li , Y. Wu , and T. Wang , “Single‐Cell Transcriptomics of Neuroinflammation and Cerebrovascular Endothelial Cells in the Aged Rat Hippocampus,” iScience 28, (2025): 113332. 40933643 10.1016/j.isci.2025.113332 PMC12419112

[22] A. P. Voigt , K. Mulfaul , N. K. Mullin , et al., “Single‐Cell Transcriptomics of the Human Retinal Pigment Epithelium and Choroid in Health and Macular Degeneration,” Proceedings of the National Academy of Sciences 116 (2019): 24100–24107. 10.1073/pnas.1914143116 PMC6883845 31712411

[23] X. Zhang , Y. Xiao , B. Hu , et al., “Multi‐Omics Analysis of Human Tendon Adhesion Reveals That ACKR1‐Regulated Macrophage Migration Is Involved in Regeneration,” Bone Research 12 (2024): 27. 38714649 10.1038/s41413-024-00324-w PMC11076548

[24] L. Zhao , S. Han , H. Su , et al., “Single‐Cell Transcriptome Atlas of the Human Corpus Cavernosum,” Nature Communications 13 (2022): 4302. 10.1038/s41467-022-31950-9 PMC9314400 35879305

[25] F. Wang , P. Ding , X. Liang , et al., “Endothelial Cell Heterogeneity and Microglia Regulons Revealed by a Pig Cell Landscape at Single‐Cell Level,” Nature Communications 13 (2022): 3620. 10.1038/s41467-022-31388-z PMC9232580 35750885

[26] J. B. Weitzman , L. Fiette , K. Matsuo , and M. Yaniv , “JunD Protects Cells from p53‐Dependent Senescence and Apoptosis,” Molecular Cell 6 (2000): 1109–1119. 11106750 10.1016/s1097-2765(00)00109-x

[27] C. Iavarone , A. Catania , M. J. Marinissen , et al., “The Platelet‐Derived Growth Factor Controls c‐myc Expression Through a JNK‐ and AP‐1‐Dependent Signaling Pathway,” Journal of Biological Chemistry 278 (2003): 50024–50030. 14523011 10.1074/jbc.M308617200

[28] Z.‐S. Zhang , Y.‐Y. Liu , S.‐S. He , et al., “Pericytes Protect Rats and Mice from Sepsis‐induced Injuries by Maintaining Vascular Reactivity and Barrier Function: Implication of miRNAs and Microvesicles,” Military Medical Research 10 (2023): 13. 36907884 10.1186/s40779-023-00442-2 PMC10010010

[29] G.‐J. Shi , Z.‐M. Li , J. Zheng , et al., “Diabetes Associated with Male Reproductive System Damages: Onset of Presentation, Pathophysiological Mechanisms and Drug Intervention,” Biomedicine & Pharmacotherapy 90 (2017): 562–574. 28407577 10.1016/j.biopha.2017.03.074

[30] L. Guariguata , D. R. Whiting , I. Hambleton , J. Beagley , U. Linnenkamp , and J. E. Shaw , “Global Estimates of Diabetes Prevalence for 2013 and Projections for 2035,” Diabetes Research And Clinical Practice 103 (2014): 137–149. 24630390 10.1016/j.diabres.2013.11.002

[31] R. Huang , J. Chen , B. Guo , C. Jiang , and W. Sun , “Diabetes‐Induced Male Infertility: Potential Mechanisms and Treatment Options,” Molecular Medicine 30 (2024): 11. 38225568 10.1186/s10020-023-00771-x PMC10790413

[32] I. F. Hall , F. Kishta , Y. Xu , A. H. Baker , and J. C. Kovacic , “Endothelial to Mesenchymal Transition: At the Axis of Cardiovascular Health and Disease,” Cardiovascular Research 120 (2024): 223–236. 38385523 10.1093/cvr/cvae021 PMC10939465

[33] D. Medici and R. Kalluri , “Endothelial–Mesenchymal Transition and Its Contribution to the Emergence of Stem Cell Phenotype,” Seminars In Cancer Biology 22 (2012): 379–384. 22554794 10.1016/j.semcancer.2012.04.004 PMC3422405

[34] S. Lovisa , E. Fletcher‐Sananikone , H. Sugimoto , et al., “Endothelial‐to‐Mesenchymal Transition Compromises Vascular Integrity to Induce Myc‐mediated Metabolic Reprogramming in Kidney Fibrosis,” Science Signaling 13 (2020): aaz2597. 10.1126/scisignal.aaz2597 PMC7790440 32518142

[35] P. Lindblom , H. Gerhardt , S. Liebner , et al., “Endothelial PDGF‐B Retention Is Required for Proper Investment of Pericytes in the Microvessel Wall,” Genes & Development 17 (2003): 1835–1840. 12897053 10.1101/gad.266803 PMC196228

[36] Q. Ma , X. He , X. Wang , et al., “PTPN14 Aggravates Neointimal Hyperplasia Via Boosting PDGFRβ Signaling in Smooth Muscle Cells,” Nature Communications 15 (2024): 7398. 10.1038/s41467-024-51881-x PMC11350182 39191789

[37] M. Zhao , L. Wang , M. Wang , et al., “Targeting Fibrosis: Mechanisms and Clinical Trials,” Signal Transduction and Targeted Therapy 7 (2022): 206. 35773269 10.1038/s41392-022-01070-3 PMC9247101

[38] T. Ostendorf , P. Boor , C. R. van Roeyen , and J. Floege , “Platelet‐Derived Growth Factors (PDGFs) in Glomerular and Tubulointerstitial Fibrosis,” Kidney International Supplements 4 (2014): 65–69. 26312152 10.1038/kisup.2014.12 PMC4536969

[39] X. Li , M. Tjwa , L. Moons , et al., “Revascularization of Ischemic Tissues by PDGF‐CC via Effects on Endothelial Cells and Their Progenitors,” Journal of Clinical Investigation 115 (2005): 118–127. 15630451 10.1172/JCI19189 PMC535797

[40] C. He , S. C. Medley , T. Hu , et al., “PDGFRβ Signalling Regulates Local Inflammation and Synergizes with Hypercholesterolaemia to Promote Atherosclerosis,” Nature Communications 6 (2015): 7770. 10.1038/ncomms8770 PMC4507293 26183159

[41] J. M. Embil , K. Papp , G. Sibbald , et al., “Recombinant Human Platelet‐Derived Growth Factor‐BB (Becaplermin) for Healing Chronic Lower Extremity Diabetic Ulcers: An Open‐Label Clinical Evaluation of Efficacy,” Wound Repair And Regeneration 8 (2000): 162–168. 10886806 10.1046/j.1524-475x.2000.00162.x

[42] X.‐H. Zhao , H.‐F. Gu , Z.‐R. Xu , et al., “Efficacy of Topical Recombinant human Platelet‐derived Growth Factor for Treatment of Diabetic Lower‐extremity Ulcers: Systematic Review and Meta‐analysis,” Metabolism 63 (2014): 1304–1313. 25060693 10.1016/j.metabol.2014.06.005

[43] L. Al‐Zube , E. A. Breitbart , J. P. O'Connor , et al., “Recombinant Human Platelet‐Derived Growth Factor BB (rhPDGF‐BB) and Beta‐Tricalcium Phosphate/Collagen Matrix Enhance Fracture Healing in a Diabetic Rat Model,” Journal Of Orthopaedic Research 27 (2009): 1074–1081. 19170096 10.1002/jor.20842

[44] W. A. Tyndall , H. A. Beam , C. Zarro , J. P. O'Connor , and S. S. Lin , “Decreased Platelet Derived Growth Factor Expression during Fracture Healing in Diabetic Animals,” Clinical Orthopaedics And Related Research 408 (2003): 319–330. 10.1097/00003086-200303000-00043 12616077

[45] J. A. Lamb , J. J. Ventura , P. Hess , R. A. Flavell , and R. J. Davis , “JunD Mediates Survival Signaling by the JNK Signal Transduction Pathway,” Molecular Cell 11 (2003): 1479–1489. 12820962 10.1016/s1097-2765(03)00203-x

[46] R. M. Perciavalle and J. T. Opferman , “Delving deeper: MCL‐1's Contributions to Normal and Cancer Biology,” Trends In Cell Biology 23 (2013): 22–29. 23026029 10.1016/j.tcb.2012.08.011 PMC3532576

[47] M. Chen , C. Chang , B. Levian , D. T. Woodley , and W. Li , “Why Are There So Few FDA‐Approved Therapeutics for Wound Healing?,” International Journal of Molecular Sciences 24 (2023): 15109. 37894789 10.3390/ijms242015109 PMC10606455

[48] J. O. Hollinger , C. E. Hart , S. N. Hirsch , S. Lynch , and G. E. Friedlaender , “Recombinant Human Platelet‐Derived Growth Factor: Biology and Clinical Applications,” Journal Of Bone And Joint Surgery‐American Volume 90 (2008): 48–54. 18292357 10.2106/JBJS.G.01231

[49] M. Nevins , W. V. Giannobile , M. K. McGuire , et al., “Platelet‐Derived Growth Factor Stimulates Bone Fill and Rate of Attachment Level Gain: Results of a Large Multicenter Randomized Controlled Trial,” Journal of Periodontology 76 (2005): 2205–2215. 16332231 10.1902/jop.2005.76.12.2205

[50] Q. Jin , O. Anusaksathien , S. A. Webb , M. A. Printz , and W. V. Giannobile , “Engineering of Tooth‐Supporting Structures by Delivery of PDGF Gene Therapy Vectors,” Molecular Therapy 9 (2004): 519–526. 15093182 10.1016/j.ymthe.2004.01.016 PMC2572773

[51] A. Kumar , J. Jovel , J. Lopez‐Orozco , et al., “Human Sertoli Cells Support High Levels of Zika Virus Replication and Persistence,” Scientific Reports 8 (2018): 5477. 29615760 10.1038/s41598-018-23899-x PMC5883016

[52] X. Nie , S. K. Munyoki , M. Sukhwani , et al., “Single‐Cell Analysis of Human Testis Aging and Correlation with Elevated Body Mass Index,” Developmental Cell 57 (2022): 1160–1176. 35504286 10.1016/j.devcel.2022.04.004 PMC9090997

[53] Y. Hao , S. Hao , E. Andersen‐Nissen , et al., “Integrated Analysis of Multimodal Single‐Cell Data,” Cell 184 (2021): 3573–3587. 34062119 10.1016/j.cell.2021.04.048 PMC8238499

[54] I. Korsunsky , N. Millard , J. Fan , et al., “Fast, Sensitive and Accurate Integration of Single‐Cell Data with Harmony,” Nature Methods 16 (2019): 1289–1296. 31740819 10.1038/s41592-019-0619-0 PMC6884693

[55] S. Jin , C. F. Guerrero‐Juarez , L. Zhang , et al., “Inference and Analysis of Cell‐Cell Communication Using CellChat,” Nature Communications 12 (2021): 1088. 10.1038/s41467-021-21246-9 PMC7889871 33597522

[56] G. Yu , L. G. Wang , Y. Han , and Q. Y. He , “clusterProfiler: An R Package for Comparing Biological Themes Among Gene Clusters,” OMICS: A Journal of Integrative Biology 16 (2012): 284–287. 22455463 10.1089/omi.2011.0118 PMC3339379

[57] B. Van De Sande , C. Flerin , K. Davie , et al., “A Scalable SCENIC Workflow for Single‐Cell Gene Regulatory Network Analysis,” Nature Protocols 15 (2020): 2247–2276. 32561888 10.1038/s41596-020-0336-2

[58] X. Qiu , A. Hill , J. Packer , D. Lin , Y. A. Ma , and C. Trapnell , “Single‐Cell mRNA Quantification and Differential Analysis with Census,” Nature Methods 14 (2017): 309–315. 28114287 10.1038/nmeth.4150 PMC5330805

[59] C.‐N. Members , “Database Resources of the National Genomics Data Center, China National Center for Bioinformation in 2022,” Nucleic Acids Research 50 (2022): D27–D38. 34718731 10.1093/nar/gkab951 PMC8728233

[60] T. Chen , X. Chen , S. Zhang , et al., “The Genome Sequence Archive Family: Toward Explosive Data Growth and Diverse Data Types,” Genomics, Proteomics & Bioinformatics 19 (2021): 578–583. 10.1016/j.gpb.2021.08.001 PMC9039563 34400360

[61] L. Cui , X. Nie , Y. Guo , et al., “Single‐Cell Transcriptomic Atlas of the Human Testis Across the Reproductive Lifespan,” Nature Aging 5 (2025): 658–674. 40033047 10.1038/s43587-025-00824-2 PMC12003174

[62] M. Wang , X. Liu , G. Chang , et al., “Single‐Cell RNA Sequencing Analysis Reveals Sequential Cell Fate Transition During Human Spermatogenesis,” Cell Stem Cell 23 (2018): 599–614. 30174296 10.1016/j.stem.2018.08.007

[63] S. Laurentino , L. Heckmann , S. Di Persio , et al., “High‐Resolution Analysis of Germ Cells from Men with Sex Chromosomal Aneuploidies Reveals Normal Transcriptome but Impaired Imprinting,” Clinical Epigenetics 11 (2019): 127. 31462300 10.1186/s13148-019-0720-3 PMC6714305

[64] L. Zhao , C. Yao , X. Xing , et al., “Single‐Cell Analysis of Developing and Azoospermia Human Testicles Reveals Central Role of Sertoli Cells,” Nature Communications 11 (2020): 5683. 10.1038/s41467-020-19414-4 PMC7655944 33173058

[65] E. Mahyari , J. Guo , A. C. Lima , et al., “Comparative Single‐Cell Analysis of Biopsies Clarifies Pathogenic Mechanisms in Klinefelter Syndrome,” The American Journal of Human Genetics 108 (2021): 1924–1945. 34626582 10.1016/j.ajhg.2021.09.001 PMC8546046

[66] M. Alfano , A. S. Tascini , F. Pederzoli , et al., “Aging, Inflammation and DNA Damage in the Somatic Testicular Niche with Idiopathic Germ Cell Aplasia,” Nature Communications 12 (2021): 5205. 10.1038/s41467-021-25544-0 PMC8410861 34471128

[67] X. Wang , Q. Liu , Z. Zhuang , et al., “Decoding the Pathogenesis of Spermatogenic Failure in Cryptorchidism through Single‐Cell Transcriptomic Profiling,” Cell Reports Medicine 5 (2024): 101709. 39226895 10.1016/j.xcrm.2024.101709 PMC11528238

