# Supplementary material for: Endothelial PDGF Signaling Dysregulation Impairs Testicular Interstitial Homeostasis in Diabetes
Source: Adv Sci (Weinh). 2026 Feb 5;13(21):e20114. doi: 10.1002/advs.202520114 (PMC13073339; doi:10.1002/advs.202520114)
Supplement: Supplementary file 1 — Supporting File 1: advs74233‐sup‐0001‐SuppMat.docx. [file ADVS-13-e20114-s002.docx]

**Figure S1 Signaling patterns and transcriptional variability of testicular cells in diabetes**

(**A**) Dot plot showing the outgoing communication patterns secreted from each testicular cell type. Dot colors represent different cell types and dot sizes represent different levels of contribution.

(**B**) Bar plot displaying the outgoing signaling strength secreted from each testicular cell type.

(**C**) Sequencing metrics of testicular samples from three DM donors.

(**D**) PCA plots before and after Harmony batch correction.

(**E**) Expression patterns of selected marker genes visualized on the UMAP embedding shown in **Figure 1F**. Expression levels are indicated by color intensity, with purple denoting high expression and gray denoting low expression, as defined in the color key at the bottom right.

(**F**) Left: Heatmap showing differentially expressed genes (DEGs) for each cell cluster identified in **Figure 1F**. Expression values are scaled to Z scores, with colors indicating relative expression levels. Right: Bar plot of Gene Ontology (GO) terms significantly enriched in the marker genes of each cluster. Bar length represents Log_10_(*p*-value), reflecting the significance of enrichment.

(**G**) Violin plot comparing gene expression variability between germ cells and somatic cells in DM versus healthy testes. The y-axis shows the Log_2_ fold change of the coefficient of variation (CV) (DM/Healthy). *****p* < 0.0001.

(**H**) Violin plots illustrating the extent of transcriptional dysregulation across testicular somatic cell populations. Each violin shows the distribution of absolute Log_2_ fold changes (Log_2_FC) in gene expression between DM and healthy testes, with horizontal bars marking the median.

(**I**) Violin plots illustrating gene expression variability in testicular somatic cell populations. The x-axis represents cell types, and the y-axis shows the Log_2_ fold change of the CV between DM and healthy testes. Colors denote cell types as indicated in the legend.

Statistical significance was determined using the Kruskal-Wallis test, and where significant, pairwise comparisons were conducted with the Wilcoxon rank-sum test, incorporating Benjamini-Hochberg correction for multiple comparisons (G, H, I).

**Figure S2 Characterization of TEC subclusters and their alterations in diabetic testes**

(**A**) Expression patterns of selected marker genes mapped on the t-SNE plot in **Figure 2A**. Purple denotes high expression and gray denotes low expression, as shown in the color key at the bottom right.

(**B**) Principal component analysis (PCA) of TECs. Each symbol represents one TEC subcluster in PC1–PC2 space.

(**C**) Violin plots showing the expression of representative TEC markers across different subclusters. Lower panels show distributions of sequencing depth (nCount_RNA) and detected features (nFeature_RNA) across different subclusters, confirming data quality.

(**D**) Representative immunofluorescence staining of ACTA2 (magenta) and CD31 (green) in testicular sections from healthy controls and DM patients. Nuclei are counterstained with DAPI (blue). Scale bars, 50 μm.

(**E**) Violin plot comparing *a*ngiogenesis scores of TECs across various disease types.

(**F**) Violin plot illustrating the expression level of *PDGFB* across different cell types in healthy samples.

(**G**) Violin plot comparing *PDGFB* expression in TECs between healthy and diabetic testes. *****p* < 0.0001.

**Figure S3 Impaired TEC-derived PDGF signaling in diabetic testes**

(**A**) Dot plot representing significantly altered ligand-receptor pairs derived from TECs to other testicular cells in healthy and DM patients. Dot sizes reflect *p* value and dot colors indicate communication probability.

(**B**) Scatter plot illustrating the outgoing interaction strength of different testicular cell types in the PDGF signaling pathway.

(**C**) Expression patterns of *PDGFB* and *PDGFRB* in healthy and DM patients presented in the form of UMAP plot. Expression levels are indicated by color intensity, with purple (upper) and red (bottom) denoting high expression and gray denoting low expression, as defined in the color key at the bottom right.

(**D**) Representative immunofluorescence staining of PDGFB (magenta) and CD31 (green) in testicular sections from healthy controls and diabetic patients. Nuclei are counterstained with DAPI (gray). Insets highlight higher magnification views of the boxed regions. Scale bars, 50 μm; 5 μm (insets).

(**E**) Quantification of overall PDGFB fluorescence intensity in testicular sections from healthy and diabetic patients. The x-axis shows sample groups, and the y-axis indicates normalized PDGFB fluorescence intensity. Bars represent mean ± SD. n = 3 independent biological replicates per group. Statistical significance was determined using the two-tailed Student’s t-test. **p* < 0.05.

**Figure S4 Transcriptomic deterioration and functional defects of LCs in diabetes**

(**A**) Pseudotime trajectory analysis of LCs from combined healthy and DM testes. Each dot represents a single cell, ordered along pseudotime progression. Color intensity reflects pseudotime, with lighter shades indicating early states and darker shades indicating later states.

(**B**) Pseudotime trajectory analysis of LCs separated by condition. Healthy (green) and DM (orange) cells are mapped onto the same trajectory.

(**C**) Violin plots showing gene set scores for the collagen catabolic process in LCs from healthy and DM groups. *****p* < 0.0001.

(**D**) Representative Masson’s trichrome staining of testicular sections from healthy and DM groups.

(**E**) Dot plot showing expression of antioxidant genes in LCs from healthy and DM testes. Dot size represents the proportion of cells expressing each gene, and color intensity reflects average expression level.

(**F**) Dot plot showing expression of apoptosis-related genes in LCs from healthy and DM testes. Dot size represents the proportion of cells expressing each gene, and color intensity reflects average expression level.

(**G**) Histogram displaying the top 20 regulons with the most overlapping DEGs. The x-axis indicates the number of overlapping DEGs, and the y-axis shows the regulons.

(**H**) GO enrichment analysis of target genes regulated by JUND in LCs. Bar length indicates –Log_10_(*p*-value), and bar color reflects gene counts.

(**I**) Violin plot showing *JUND* expression levels in LCs from healthy and DM testes. *****p* < 0.0001.

(**J**) Dot plot showing the expression of apoptosis-related genes in LCs from healthy and DM testes. Dot size indicates the proportion of cells expressing the gene, and color intensity represents average expression level.

(**K**) Violin plot showing the expression of *MCL1* in LCs from healthy and DM testes. Each dot represents a single cell. **p* < 0.05.

(**L**) Representative immunofluorescence staining of CYP11A1 (green) or CYP17A1 (green) and DAPI (blue) in cultured human primary LCs. Scale bars, 50 μm.

(**M**) Western blot analysis of JUND and MCL1 in human primary LCs transfected with control siRNA (siNC) or JUND-targeting siRNA (siJUND). JUND and MCL1 protein levels were normalized to GAPDH and expressed as fold changes relative to the control group (siNC). Data are presented as mean ± SD. n = 3 independent biological replicates. Statistical significance was determined using the two-tailed Student’s t-test. ***p* < 0.01, ****p* < 0.001.

(**N**) Relative mRNA expression of *MCL1* in human primary LCs following JUND knockdown. Data are presented as mean ± SD. n = 3 independent biological replicates. Statistical significance was determined using the two-tailed Student’s t-test. **p* < 0.05.

**Figure S5 Diabetes-associated transcriptomic alterations in TPCs**

(**A**) Violin plots showing the expression of collagen-related ECM genes in TPCs from healthy and DM testes. ****p* < 0.001.

(**B**) Dot plot showing the expression of anti-apoptotic genes (*XIAP*, *MCL1*, *BCL2*) and pro-apoptotic genes (*FAS*, *CYCS*) in TPCs from healthy and DM testes. Dot size represents the proportion of cells expressing each gene, and color intensity indicates the average expression level.

(**C-D**) Violin plots showing the expression of contractility-associated genes in TPCs from healthy and DM testes. ****p* < 0.001.

**Figure S6 Impact of PDGFBB supplementation on interstitial homeostasis**

(**A-C**) RT-PCR analysis of *RAF1,* *BCL2,* and *CDK4* expression in *ex vivo* cultured diabetic human testicular tissue with or without PDGFBB supplementation. Bars represent mean ± SD. *****p* < 0.0001, **p* < 0.05.

(**D**) Representative immunofluorescence staining of INSL3 (magenta), Collagen III (green) and TUNEL (yellow) in *ex vivo* cultured diabetic human testicular tissue with or without PDGFBB supplementation from sample #2. Nuclei are counterstained with DAPI (blue). Insets show magnified views. Scale bars, 10 μm.

(**E**) Quantification of LC area relative to total tissue area in *ex vivo* cultured diabetic human testicular tissue with or without PDGFBB supplementation. Bars represent mean ± SD. n = 3 independent biological replicates per group. **P* < 0.05.

(**F**) Proportion of apoptotic LCs in *ex vivo* cultured diabetic human testicular tissue with or without PDGFBB supplementation. Bars represent mean ± SD. n = 3 independent biological replicates per group. **P* < 0.05.

(**G**) Working model. In DM LCs, the PDGF pathway was disrupted, and the expression of JUND and MCL1 was downregulated, which was associated with LC apoptosis, and accompanied by reduced testosterone secretion and increased ECM deposition. PDGFBB supplementation partially restored JUND and MCL1 expression, mitigated apoptosis, improved testosterone secretion, and reduced ECM deposition.

(**H**) Representative immunofluorescence staining of JUND (magenta) and CYP17A1 (green) in *ex vivo* cultured testicular biopsies from healthy donors, maintained as control or treated with PDGFBB (100 ng/mL) in the presence or absence of the PDGFRB inhibitor CP-673451 (10 μM). Nuclei are counterstained with DAPI (blue). Scale bars, 5 μm.

(**I**) Representative immunofluorescence staining of MCL1 (magenta) and CYP11A1 (green) in *ex vivo* cultured testicular biopsies from healthy donors, maintained as control or treated with PDGFBB (100 ng/mL) in the presence or absence of the PDGFRB inhibitor CP-673451 (10 μM). Nuclei are counterstained with DAPI (blue). Scale bars, 5 μm.

(**J**) Bar graph showing testosterone concentrations measured by ELISA in supernatants from ex vivo cultured testicular biopsies from healthy donors. Samples were maintained as control or treated with PDGFBB (100 ng/mL) in the presence or absence of the PDGFRB inhibitor CP-673451 (10 μM). Bars represent mean ± SD. ***p* < 0.01, ****p* < 0.001.

Statistical significance was determined by two-tailed Student’s t-test (E, F) or one-way ANOVA with Tukey’s post hoc test (J).
