# Supplementary material for: Endothelial PDGF Signaling Dysregulation Impairs Testicular Interstitial Homeostasis in Diabetes
Source: Adv Sci (Weinh). 2026 Feb 5;13(21):e20114. doi: 10.1002/advs.202520114 (PMC13073339; doi:10.1002/advs.202520114)
Supplement: Supplementary file 2 — Supporting File 2: advs74233‐sup‐0002.FigS1.pdf. [file ADVS-13-e20114-s006.pdf]

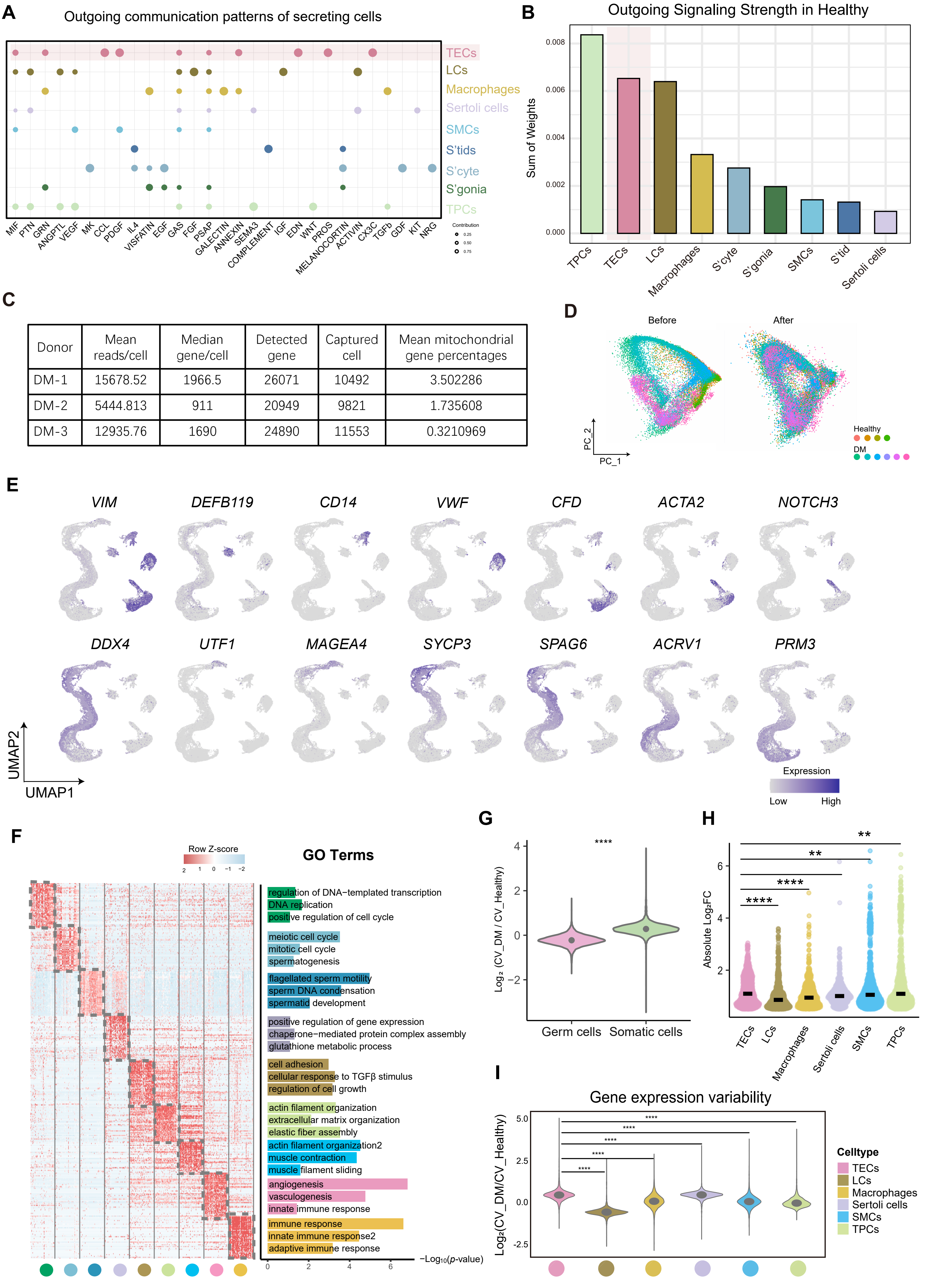

Donor	Mean reads/cell	Median gene/cell	Detected gene	Captured cell	Mean mitochondrial gene percentages
DM-1	15678.52	1966.5	26071	10492	3.502286
DM-2	5444.813	911	20949	9821	1.735608
DM-3	12935.76	1690	24890	11553	0.3210969
