# Supplementary figures and images for: Endothelial PDGF Signaling Dysregulation Impairs Testicular Interstitial Homeostasis in Diabetes
Source: Adv Sci (Weinh). 2026 Feb 5;13(21):e20114. doi: 10.1002/advs.202520114 (PMC13073339; doi:10.1002/advs.202520114)

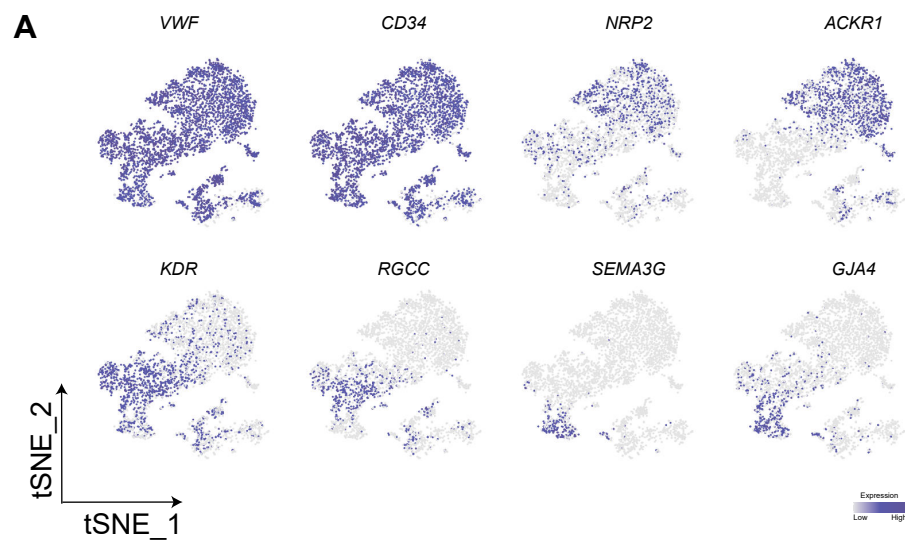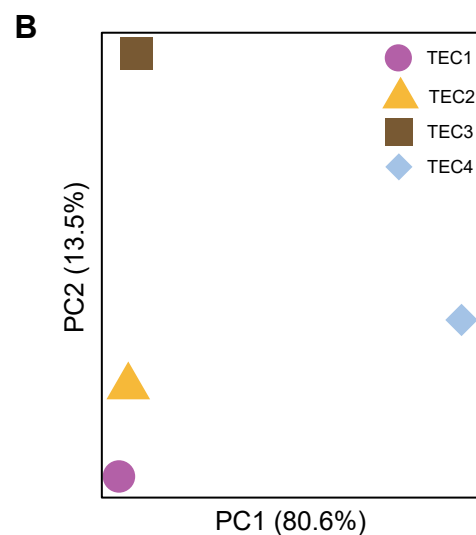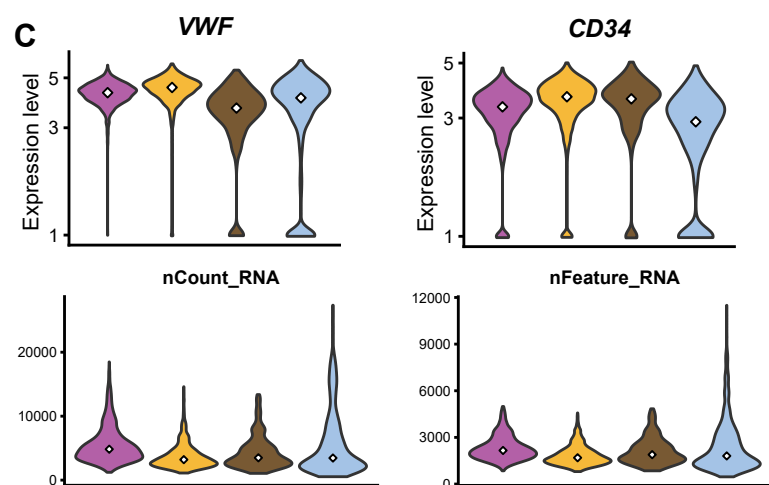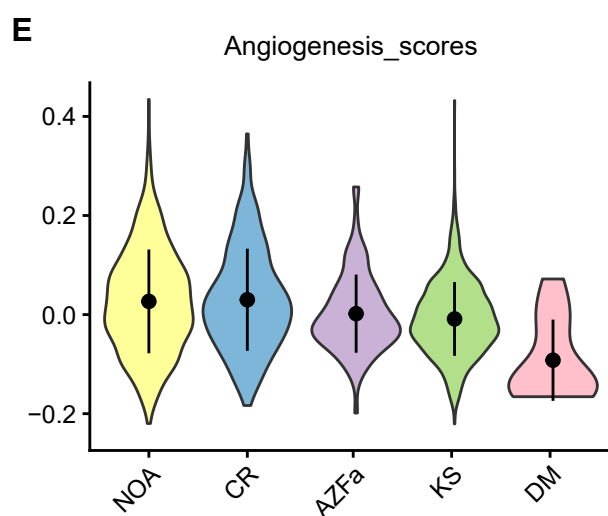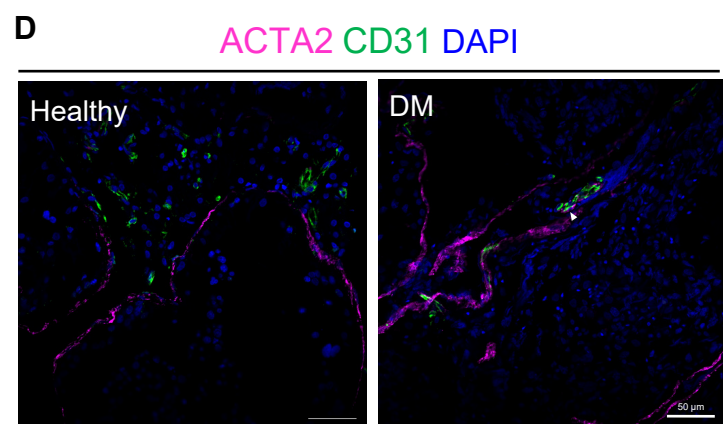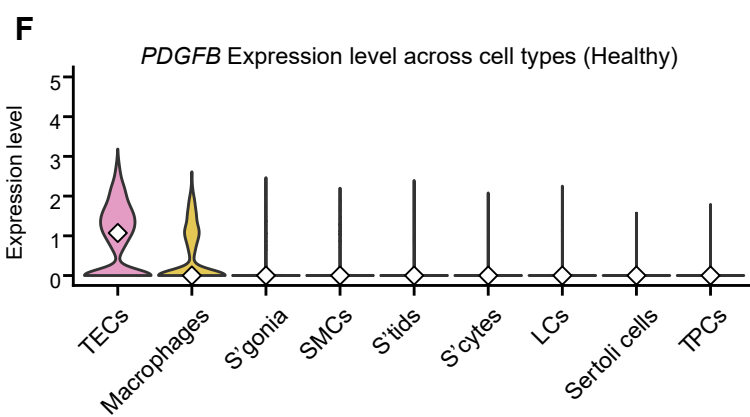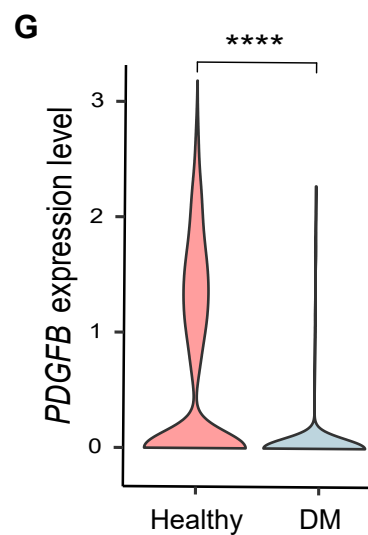

Supplement: Supplementary file 3 — Supporting File 3: advs74233‐sup‐0003.FigS2.pdf. [file ADVS-13-e20114-s008.pdf]

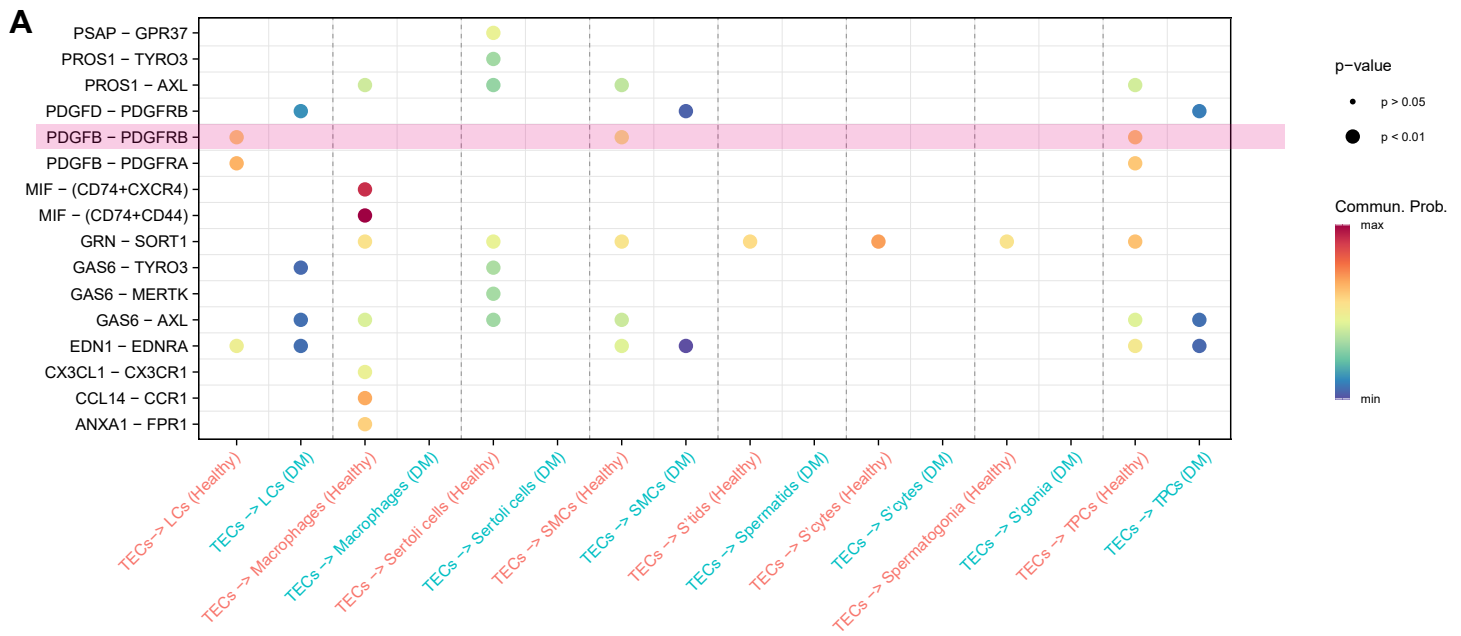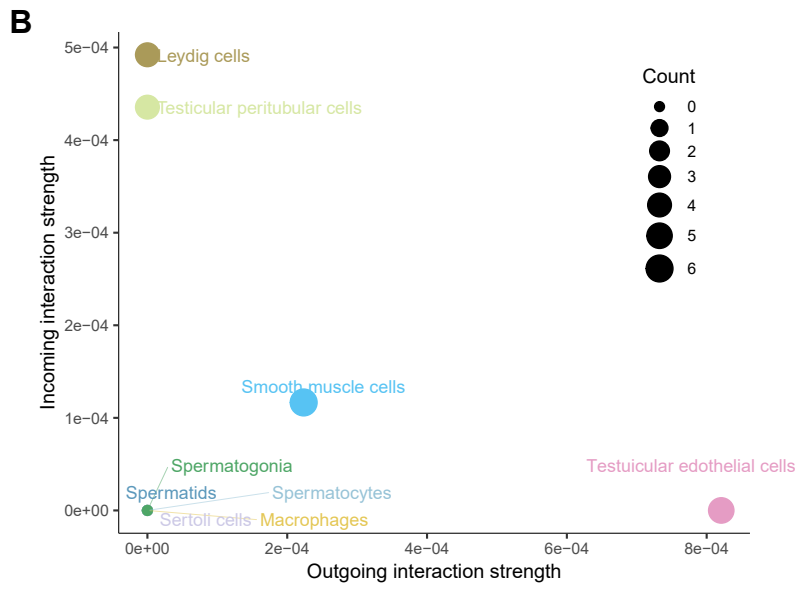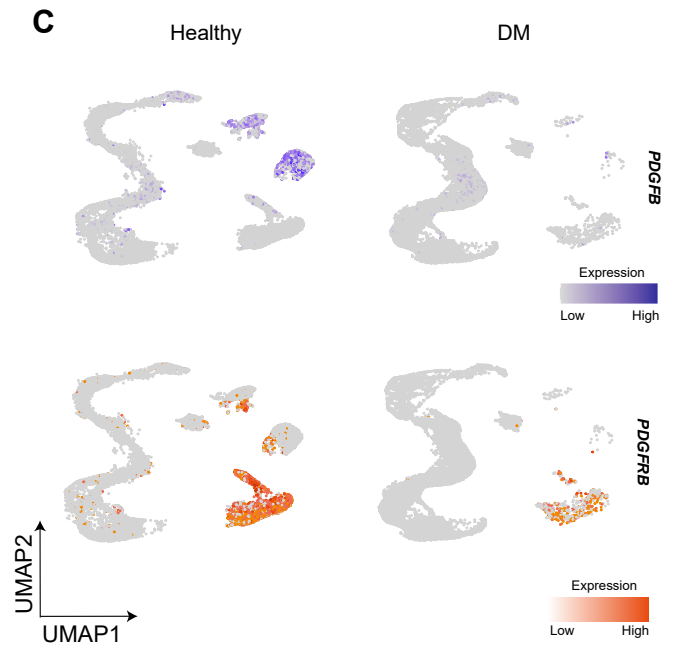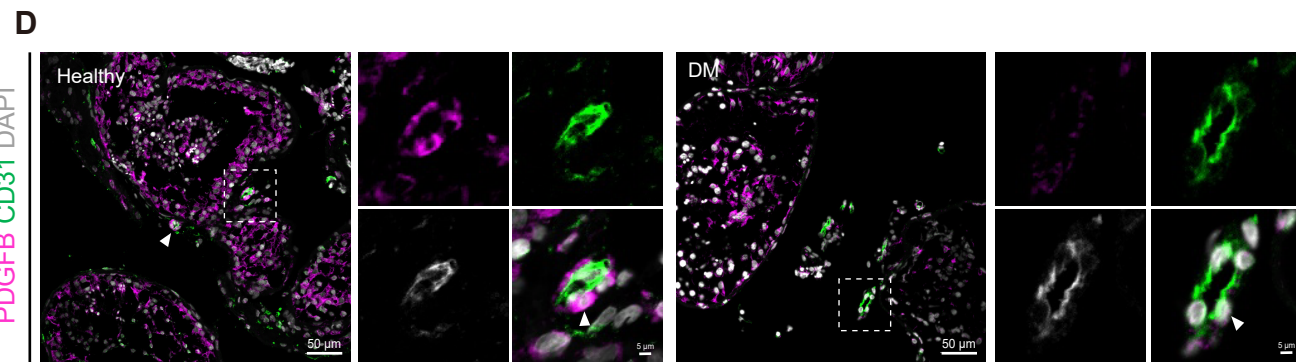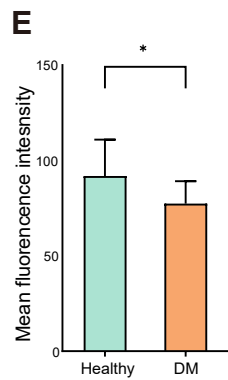

Supplement: Supplementary file 4 — Supporting File 4: advs74233‐sup‐0004.FigS3.pdf. [file ADVS-13-e20114-s003.pdf]

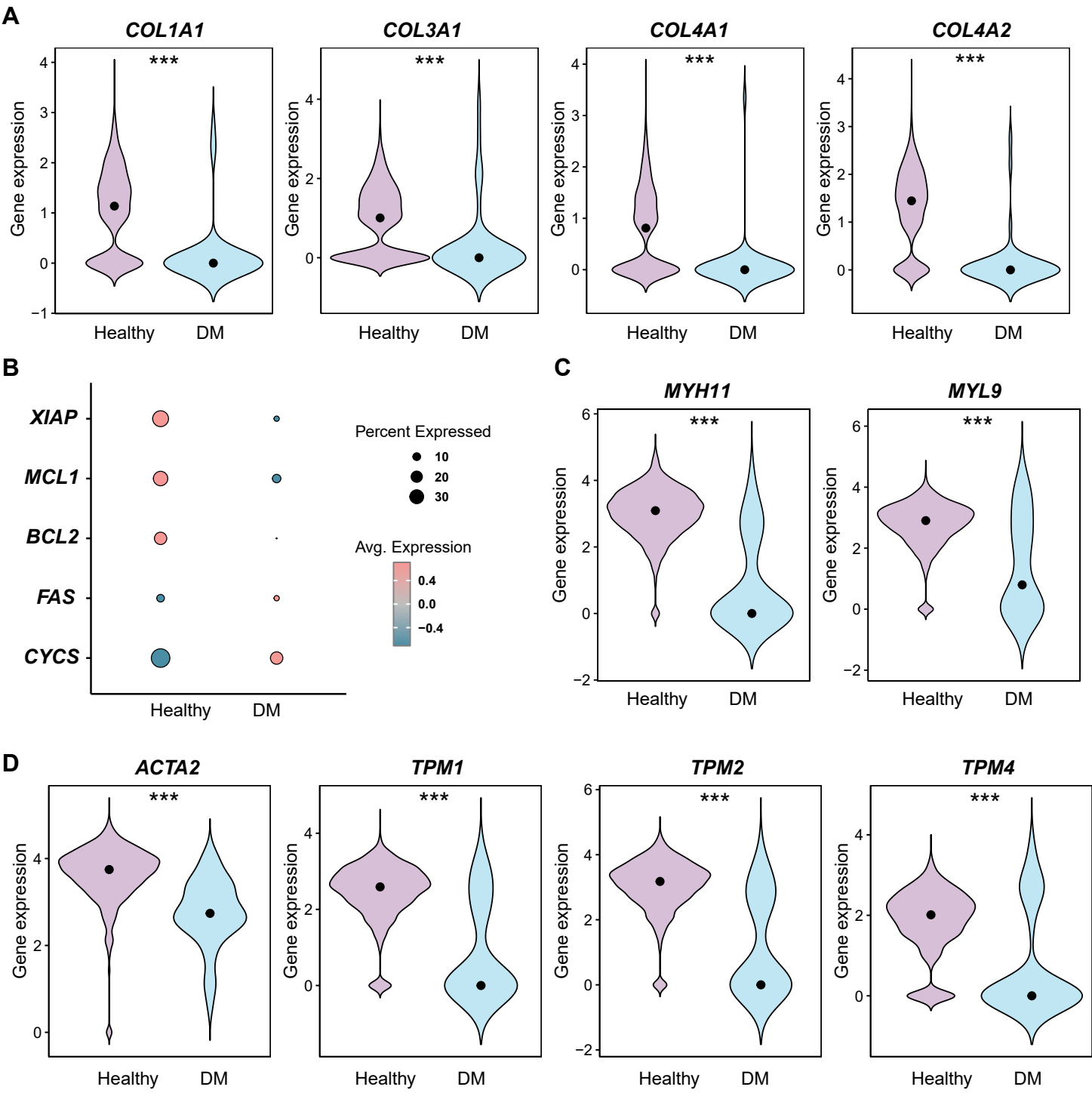

Supplement: Supplementary file 6 — Supporting File 6: advs74233‐sup‐0006.FigS5.pdf. [file ADVS-13-e20114-s001.pdf]

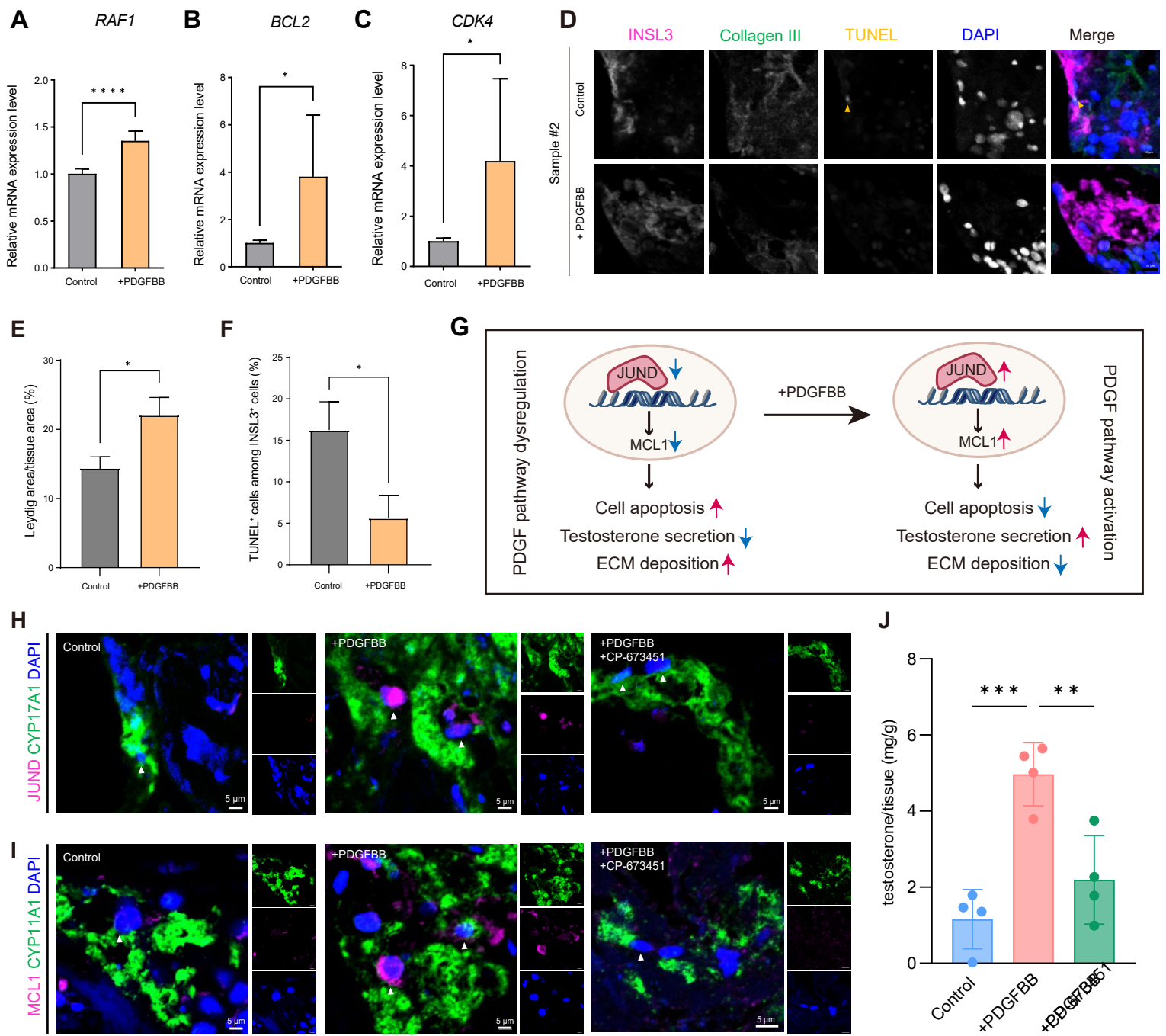

Supplement: Supplementary file 7 — Supporting File 7: advs74233‐sup‐0007.FigS6.pdf. [file ADVS-13-e20114-s007.pdf]
