# Supplementary material for: Endothelial PDGF Signaling Dysregulation Impairs Testicular Interstitial Homeostasis in Diabetes
Source: Adv Sci (Weinh). 2026 Feb 5;13(21):e20114. doi: 10.1002/advs.202520114 (PMC13073339; doi:10.1002/advs.202520114)
Supplement: Supplementary file 5 — Supporting File 5: advs74233‐sup‐0005.FigS4.pdf. [file ADVS-13-e20114-s005.pdf]

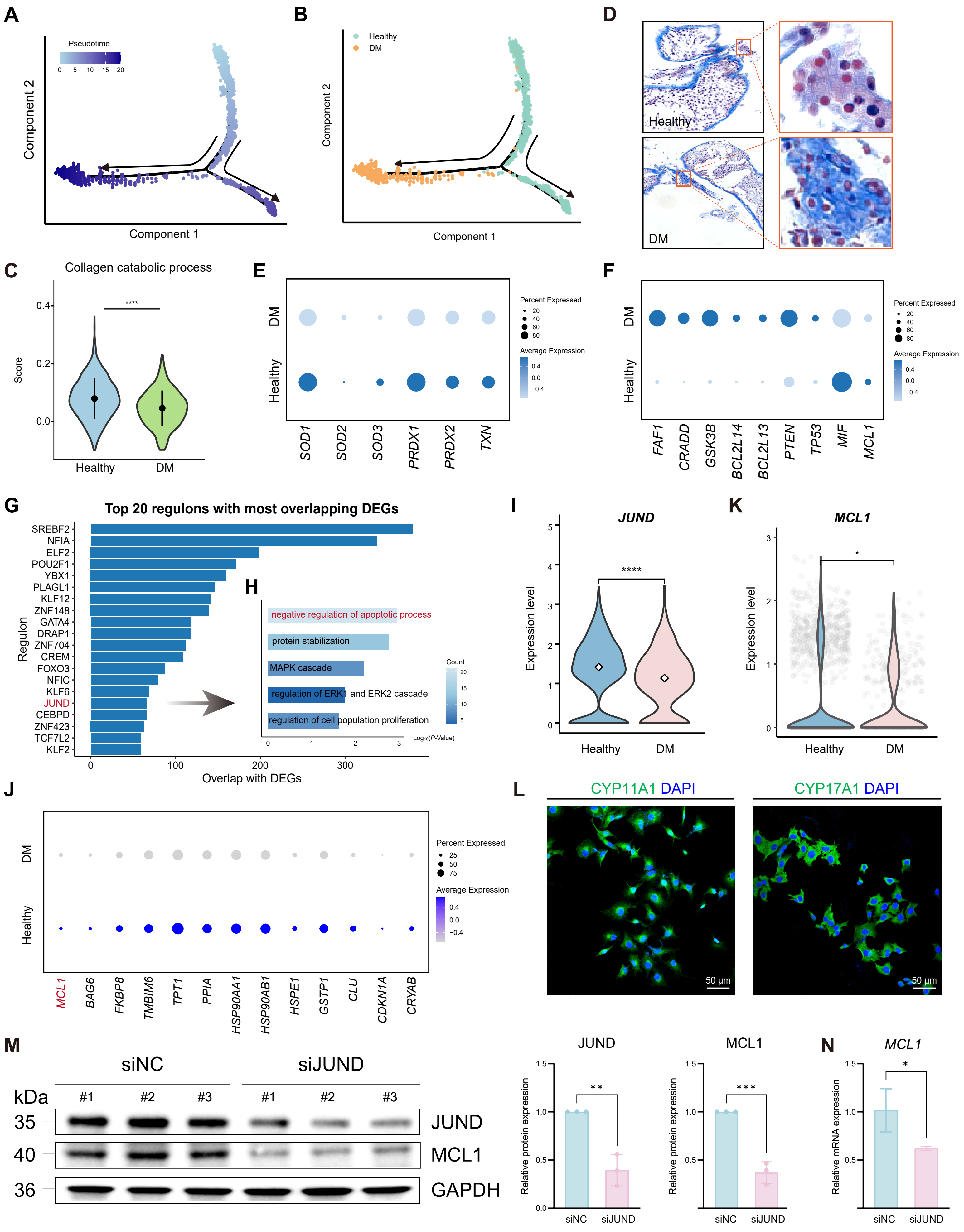

A
Pseudotime
0 5 10 15 20
Component 2
Component 1
B
Healthy
DM
Component 2
Component 1
D
Healthy
DM
C
Collagen catabolic process
Score
****
Healthy
DM
E
Percent Expressed
20
40
60
80
Average Expression
0.4
0.0
−0.4
DM
Healthy
SOD1
SOD2
SOD3
PRDX1
PRDX2
TXN
F
Percent Expressed
Average Expression
DM
Healthy
FAF1
CRADD
GSK3B
BCL2L14
BCL2L13
PTEN
TP53
MIF
MCL1
G
Top 20 regulons with most overlapping DEGs
Regulon
SREBF2
NFIA
ELF2
POU2F1
YBX1
PLAGL1
KLF12
ZNF148
GATA4
DRAP1
ZNF704
CREM
FOXO3
NFIC
KLF6
JUND
CEBPD
ZNF423
TCF7L2
KLF2
Overlap with DEGs
H
negative regulation of apoptotic process
protein stabilization
MAPK cascade
regulation of ERK1 and ERK2 cascade
regulation of cell population proliferation
Count
−Log10(P-Value)
I
JUND
Expression level
****
Healthy
DM
K
MCL1
Expression level
*
Healthy
DM
J
Percent Expressed
25
50
75
Average Expression
0.4
0.0
−0.4
DM
Healthy
MCL1
BAG6
FKBP8
TMBIM6
TPT1
PPIA
HSP90AA1
HSP90AB1
HSPE1
GSTP1
CLU
CDKN1A
CRYAB
L
CYP11A1 DAPI
CYP17A1 DAPI
50 μm
50 μm
M
siNC
siJUND
kDa
#1
#2
#3
#1
#2
#3
35
40
36
JUND
MCL1
GAPDH
JUND
MCL1
Relative protein expression
**
***
siNC
siJUND
N
MCL1
Relative mRNA expression
*
siNC
siJUND
